# More knowledge causes a focused attention deployment pattern leading to lower creative performances

**DOI:** 10.1038/s41598-021-97215-5

**Published:** 2021-09-14

**Authors:** Kunhao Yang, Itsuki Fujisaki, Kazuhiro Ueda

**Affiliations:** 1 Graduate School of Arts and Sciences, The University of Tokyo, Tokyo, 153-8902 Japan; 2 Research Fellowship for Young Scientists (DC2), Japan Society for the Promotion of Science (JSPS), Tokyo, 102-0083 Japan

**Keywords:** Attention, Problem solving, Human behaviour

## Abstract

Previous studies demonstrate that people with less professional knowledge can achieve higher performance than those with more professional knowledge in creative activities. However, the factors related to this phenomenon remain unclear. Based on previous discussions in cognitive science, we hypothesised that people with different amounts of professional knowledge have varying attention deployment patterns, leading to different creative performances. To examine our hypothesis, we analysed two datasets collected from a web-based survey and a popular online shopping website, Amazon.com (United States). We found that during information processing, people with less professional knowledge tended to give their divided attention, which positively affected creative performances. Contrarily, people with more professional knowledge tended to give their concentrated attention, which had a negative effect. Our results shed light on the relation between the amount of professional knowledge and attention deployment patterns, thereby enabling a deeper understanding of the factors underlying the different creative performances of people with varying amounts of professional knowledge.

## Introduction

Creative activity has long been considered as among the factors of development of modern society^1^. Although specific definitions of creative activity differ depending on disciplines, there is a consensus to broadly define it as an activity that generates new information combinations^1^. Based on this definition, many previous studies^2–7^ have discussed one of the core issues of creative activity: what factors can cause participants’ high *creative* *performances* (i.e., achievements of novel information combinations) in creative activities. To address this issue, previous studies^2–7^ have pointed out professional knowledge (i.e., the expertise in the corresponding domain) as among the key factors. These studies have focused on technological breakthroughs in industries and scientific breakthroughs. They have found that these breakthroughs were mainly performed by a small group of specialists who had a large amount of professional knowledge in the corresponding field. They then have concluded that professional knowledge serves as an important *information* *basis* for creative activities. In other words, people’s high creative performances are constructed based on their professional knowledge.

However, because of the dramatic development of the internet in recent decades, people with less professional knowledge have begun to participate in creative activities (e.g., the crowd-sourcing innovation of new products)^8–11^. Many recent studies^8–11^ have found that these people can also achieve high performances in creative activities. According to these studies, in creative activities, *regular* *users* on the customer-side who have almost no professional knowledge can achieve higher creative performances than *specialists* on the manufacturer side who have extensive professional knowledge. Based on the above discussions of professional knowledge^2–7^, the former can be considered as lacking the information bases necessary to achieve high creative performances. Therefore, understanding why people with less professional knowledge can achieve significantly high performances in creative activities is important.

Many previous studies in cognitive science^12–16^ have found that creative performance is largely affected by the *attention* *deployment* *pattern*. Attention is considered as a cognitive resource that can be allocated for human information processing^17,18^. It enables the detection, filtering, and comprehension of information. People faced with different tasks employ different deployment patterns of attention^17,18^. Typically, when there is only one primary target (i.e., one operation or one piece of information) to be processed in the task, they tend to employ the focused attention deployment pattern (or selective attention) to focus their attention on the primary target over other targets. By contrast, when there are multiple targets to be processed simultaneously, they tend to employ the divided attention deployment pattern (or distributed attention) to divide their attention among all the targets. Additionally, when the task involves consistent information processing, the sustained attention deployment pattern is used to maintain the attention deployment for a long period. Based on these different attention deployment patterns, previous studies^19–22^ have found that in creative activities, people who employ the divided attention deployment pattern can achieve higher creative performances than those who employ the focused attention deployment pattern. This is because people dividing their attention among multiple targets in creative activities can have more chances of finding divergent information combinations, which can serve as the bases of their high creative performances^12,13^.

Based on the above discussion on attention deployment patterns, this study built two *research* *hypotheses* to address why people with less professional knowledge can achieve significantly high performances in creative activities:

(1) People with different amounts of professional knowledge have different attention deployment patterns. People with more professional knowledge have a focused attention deployment pattern during information processing, whereas those with less professional knowledge have a divided attention deployment pattern.

(2) This difference in attention deployment patterns causes varying performances in creative activities. The focused attention deployment pattern undermines the creative performances of people with more professional knowledge, whereas the divided attention deployment pattern benefits the creative performances of those with less professional knowledge.

To empirically examine the above hypotheses, we constructed metrics using computer-vision and natural-language-processing methods in computer science. In the sections below, we explain our datasets and the construction of these metrics and present the empirical examination of the hypotheses.

Overall, this study uncovered the relation between the amount of professional knowledge and attention deployment patterns. It also demonstrated that attention deployment patterns can form a cognitive basis for the different creative performances of people with varying amounts of professional knowledge.

## Results

### Overview of datasets and main metrics

We gathered two datasets: a web-based survey data for Japanese participants (main dataset) and the Amazon review data for American participants (supplementary dataset). In both datasets, we used an audio speaker (hereafter, referred to as ‘speaker’) as a stimulus and compared the attention deployment patterns of participants with varying amounts of professional knowledge and creative performances. We gathered these two datasets from two countries because previous research^23,24^ has found that cultural background (i.e., East Asia *vs.* West) could affect participants’ attention deployment patterns.

Using these two datasets, we employed computer science methods to construct metrics of participants’ amounts of professional knowledge, attention deployment patterns, and creative performances for testing the research hypotheses. The computations of these metrics are summarised in Table 1, followed by concise explanations of these metrics.

**Table 1:** Summary of main metrics.

Dataset	Metrics of the amount of professional knowledge	Metrics of attention deployment patterns	Metrics of creative performances
Survey data	**Knowledgetestscores** based on a knowledge test in the survey i.e., the sum of the correct scores answered by a participant in the knowledge test (S2 in Supplementary Information)	**Arearatio** based on the pictures uploaded by participants in the survey and on Amazon i.e., the ratio between the area of a speaker shown in the picture and the size of the whole picture (Computation of area ratio in "Materials and methods")	**Ideanoveltytoindividuals** based on ideas submitted by participants in the survey i.e., the median of the evaluation scores of every idea (Computation of idea novelty to individuals in "Materials and methods")
			**Ideanoveltytoagroup** based on ideas submitted by participants in the survey i.e., ${\sum }_{\mathrm{w}\in \mathrm{d}}{\mathrm{Tfidf}}_{\mathrm{w}} = {\sum }_{\mathrm{w}\in \mathrm{d}}{\mathrm{p}}_{\mathrm{w},\mathrm{d}}\times \mathrm{log}({\frac{\left|\mathrm{D}\right|}{\mathrm{f}_{\mathrm{w},\mathrm{D}}}})$ (Computation of idea novelty to a group and review novelty to a group in "Materials and methods")
			**Ideanoveltyinhistory** based on ideas submitted by participants in the survey i.e., ${\sum }_{\mathrm{w}\in \mathrm{d}}{\mathrm{CB}}_{\mathrm{w}} =-{\sum }_{\mathrm{w}\in \mathrm{d}}{\mathrm{p}}_{\mathrm{w},\mathrm{d}}\times \mathrm{log}({\mathrm{p}}_{\mathrm{w},\mathrm{Q}})$ (Computation of idea novelty in history and review novelty in history in "Materials and methods")
*Amazonreviewdata*	***Distancetothespecialists****basedonreviewtextinAmazon* *i.e.,theaveragecosinedistanceofAmazonreviewstotheknownspecialists’reviews* *(ComputationofthedistancetotheknownspecialistsbasedontheAmazonreviewdatain* "Materials and methods")		***Reviewnoveltytoagroup****basedonreviewtextsinAmazon* *i.e.,*${\sum }_{w\in r}Tfid{f}_{r} = {\sum }_{w\in r}{p}_{w,d}\times log({\frac{\left|R\right|}{f_{w,R}}})$ *(ComputationofideanoveltytoagroupandreviewnoveltytoagroupinMaterialsandmethods)*
			***Reviewnoveltyinhistory****basedonreviewtextsinAmazon* *i.e.,*${\sum }_{w\in r}C{B}_{r} =-{\sum }_{w\in r}{p}_{w,r}\times log({p}_{w,Q})$ *(Computationofideanoveltyinhistoryandreviewnoveltyinhistoryin* "Materials and methods")

In this table, every metric’s name is in bold, followed by the specific data that were used for its construction and a concise explanation of its computation. Parentheses show the sections where the details of the computation were explained. The details of the area in italics are explained in the Supplementary Information.

**Figure 1:**
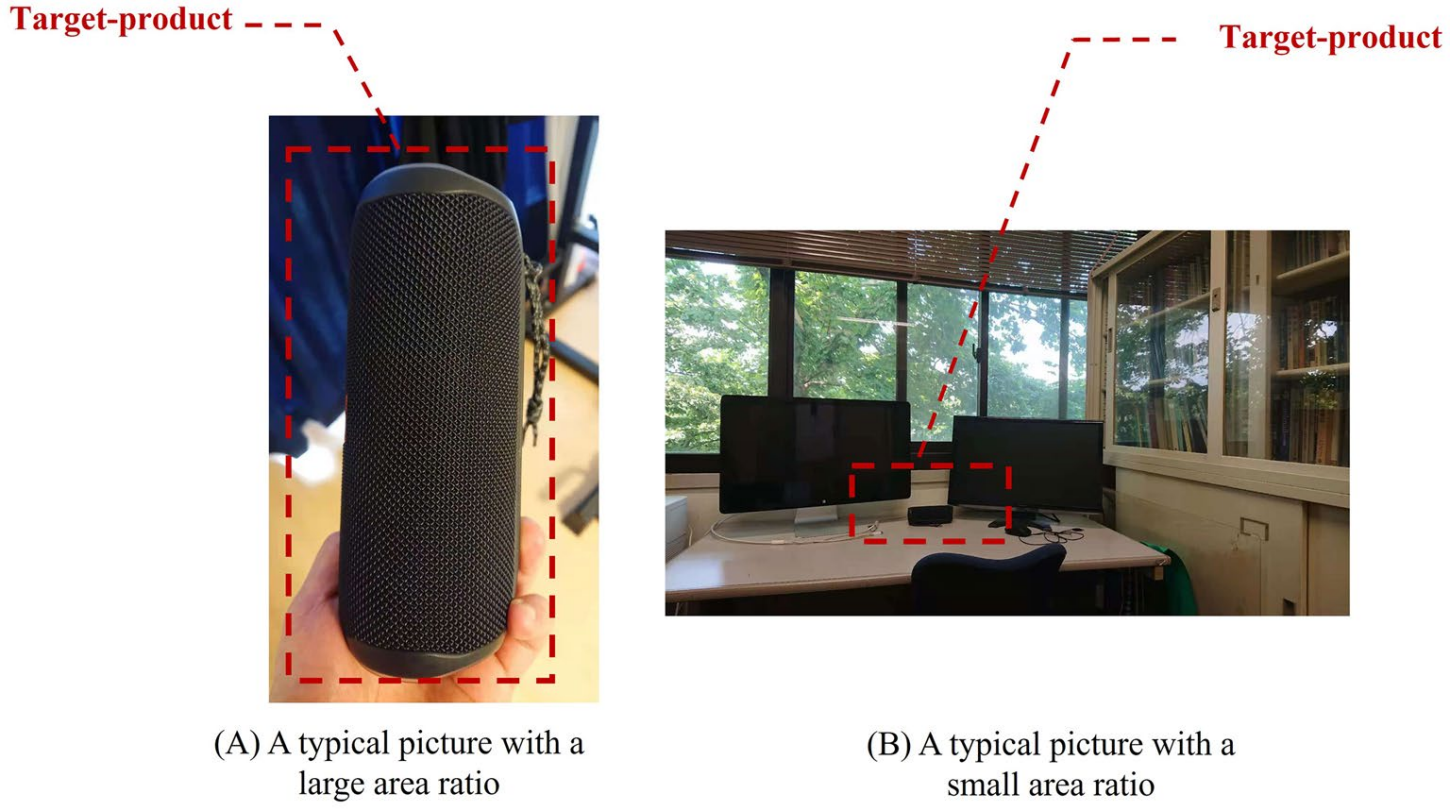
Examples of pictures with different area ratios. **(a,b)** show two examples with different area ratios. The area ratio in **(a)** is evidently larger than that in **(b)**. Therefore, we considered that a participant with a focused attention deployment pattern would submit a picture similar to **(a)**; by contrast, a participant with a divided attention deployment pattern would submit a picture similar to **(b)**. The two pictures used in this figure were taken by the first author. Therefore, these were only used for illustration but not real examples in our two datasets.

In the web-based survey, we gathered data from 200 participants (see details in Description of survey and Amazon datasets in "Materials and methods"). *To* *measure* *participants’* *amounts* *of* *professional* *knowledge*, we conducted a knowledge test on the speakers (see details in Section S2 of the Supplementary Information). Participants with higher (lower) scores in the knowledge test were considered to have more (less) professional knowledge of speakers. To measure participants’ attention deployment patterns, every participant in the survey was required to submit a picture to introduce their speakers. Previous research^12,16,17^ has found that participants’ attention deployment patterns could be reflected by these pictures. Specifically, if participants give their concentrated attention to a target product (i.e., the speaker), then the picture will not include much contextual information on other objects around the product. However, if the attention is divided, then the picture taken by the participant will include more contextual information (see an example in Fig. 1). Therefore, *to* *measure* *participants’* *attention* *deployment* *patterns*, we computed the *area* *ratio* of the target product in the picture (hereafter, referred to as the ‘area ratio’) by combining two computer-vision models^25–27^: *Yolov3-Mobilenet* and *Grabcut* (see details in "Materials and methods"). The area ratio was equal to the ratio between the size of the target product in the picture and that of the entire picture. Based on previous research^12,16,17^, participants with larger area ratios have been considered to have more focused attention deployment patterns, whereas those with smaller area ratios have more divided attention deployment patterns. In Section S3.2 of the Supplementary Information, an additional analysis tested the robustness of this metric. To measure participants’ creative performances, every participant in the survey was required to submit an idea about the new functions of future speakers. Based on these ideas, we employed the idea novelty (1) *to* *individuals*, (2) *to* *a* *group*, and (3) *in* *history* as metrics of participants’ creative performances^28,29^. The idea novelty to individuals indicated the novelty of an idea that was evaluated by a small number of experts. As the experts’ evaluations can be affected by their subjective judgements and individual backgrounds^28–30^, we also employed methods in information theory to generate the other two metrics based on computations instead of individual evaluations. Based on the methods in information theory, the idea novelty to a group reflected an idea’s novelty compared with other ideas under evaluation, whereas the idea novelty in history is an idea’s novelty compared with related information in history. Fig. S1 in the Supplementary Information (see specific correlations in the first row and second column, first row and third column, and second row and third column) shows that correlations among the above three metrics were significantly positive (idea novelty to individuals *vs.* idea novelty to a group: *correlation* = 0.22, *p* *value* = 0.02; idea novelty to individuals *vs.* idea novelty in history: *correlation* = 0.28, *p* *value* < 0.01; idea novelty to a group *vs*. idea novelty in history: *correlation* = 0.96, *p* *value* < 0.01). Since for all three metrics, larger values indicated participants’ higher creative performances, these positive correlations indicated that the three metrics’ measurements of creative performances were consistent.

The Amazon review data included 201,489 reviews and 9257 uploaded pictures of speakers. *To* *measure* *participants’* *amounts* *of* *professional* *knowledge*, we analysed their Amazon review texts. Previous studies^31–33^ have found that the similarity of the review contents could effectively reflect the similarity of the reviewers’ knowledge background. Therefore, using a document embedding model^34^, we computed every Amazon review text’s distance to some known specialists’ reviews (hereafter, referred to as the *distance* *to* *the* *specialists*; see details of the known specialists in *Computation of the distance to the known specialists based on the Amazon review data* in "Materials and methods") to indicate their amount of professional knowledge. Based on previous research^31–33^, Amazon participants who had a smaller (larger) distance to the specialists were considered as participants with more (less) professional knowledge. *To* *measure* *Amazon* *participants’* *attention* *deployment* *patterns*, we analysed the uploaded pictures and computed the same metric as in the survey data: the area ratio. As explained above, a larger (smaller) area ratio indicates a more focused (divided) attention deployment pattern. *To* *measure* *participants’* *creative* *performances,* we employed methods in information theory to compute their *review* *novelty* *to* *a* *group* and *in* *history* based on their review texts^28,29^. Review novelty to a group refers to one review’s novelty compared with others under evaluation. Review novelty in history refers to one review’s novelty compared with related information in history. Fig. S4 in the Supplementary Information (see specific correlations in the first row and second column) shows that the correlation between these two metrics was significantly positive (*correlation* = 0.79, *p* *value* < 0.01). Since for both metrics, larger values indicate participants’ higher creative performances, this positive correlation indicated that the two metrics’ measurements of creative performances were consistent. We did not compute the *review* *novelty* *to* *individuals* because its computation would have been time-intensive and it is less important for creative performance evaluation^28,29^.

The details of the above computations can be found in "Materials and methods". Finally, since the survey data were more informative for discussing participants’ creative performances, the Amazon review data were only used as a supplementary dataset. In the following analyses, we first tested our research hypotheses using the survey data to build models where the related variables suggested by the theories were controlled comprehensively. Subsequently, we employed the Amazon data to show the robustness of our research hypotheses based on a large dataset (the results were reported briefly and the details can be found in the Supplementary Information). By testing the consistency between the results based on these two datasets, we could examine the robustness of our results.

### Different attention deployment patterns among people with different amounts of professional knowledge

**Figure 2:**
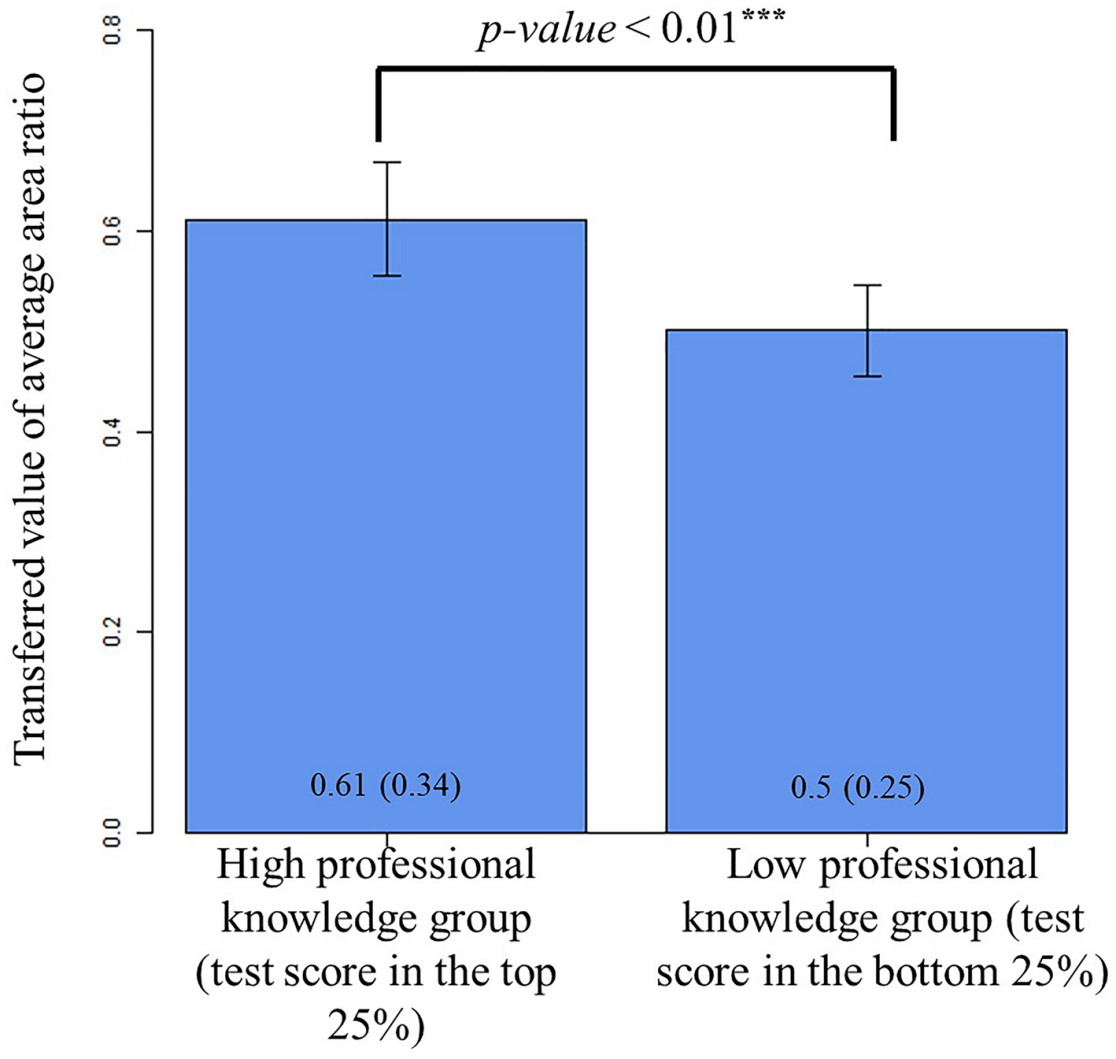
Illustration of the average area ratio of the high and low professional knowledge groups in the survey data. The left bar shows the average area ratio of the high professional knowledge group (i.e., participants with the top 25% of the test scores) while the right, that of the low professional knowledge group (i.e., participants with the bottom 25% of the test scores). The numbers inside each bar represent transformed values of the average area ratios under the arcsine transformation. The numbers inside the parentheses represent the raw average area ratios before the transformation.

We first examined the different attention deployment patterns among participants with varying amounts of professional knowledge. In Fig. 2, we used *t*-test to compare the area ratios between the participants with the top 25% test scores (i.e., the high professional knowledge group) and those with the bottom 25% test scores (i.e., the low professional knowledge group) in the survey data. As the area ratios ranged from 0 to 1, we transformed the area ratio by the arcsine transformation (i.e., ${x}_{new}=arcsine({x}^{2})$) before conducting *t-*test. As shown in Fig. 2, we found the high professional knowledge group with a significantly larger average area ratio than the low professional knowledge group (the average area ratios were 0.34 and 0.25 in the high and low professional knowledge groups, respectively; *t* = 3.07, *p* value < 0.01; *r* = 0.29).

Moreover, we employed regression models to examine the relation between the amount of professional knowledge and attention deployment patterns. Table 2 shows the regression results for the relation between participants’ test score and the area ratio in their pictures. Since the area ratio ranged from 0 to 1, we used beta-regression to build our models^35^. Because previous studies^36,37^ have found that a participant’s gender and age also affect the attention deployment pattern, we controlled participants’ gender and age in Model 1. In Model 2, we controlled participants’ basic information on speaker usage, which included the (1) number of speaker holdings, (2) frequency of speaker usage, (3) self-evaluation of the particularities of speakers, and (4) self-evaluation of the amount of professional knowledge about speakers (the details of these variables can be found in S1 and S2 sections in theSupplementary Information). Table 2 shows that in both models, participants’ test score has a significant positive effect on the target product’s area ratio in their pictures (Model 1: *coefficient* = 0.044, *p* value = 0.002; Model 2: *coefficient* = 0.051, *p* value = 0.001). These results indicated that participants with more professional knowledge of the target product submitted pictures with larger area ratios.

**Table 2:** Regression results for the area ratio of the target product in the survey data.

Dependent variable	Area ratio	
	Model 1	Model 2
Test score	0.044** (0.014)	0.051*** (0.016)
Gender	0.080 (0.172)	− 0.030 (0.173)
Age	− 0.007 (0.005)	− 0.008 (0.005)
Number of speaker holdings	−	0.011(0.016)
Frequency of speaker usage	−	− 0.074** (0.032)
Self-evaluation of the particularities of speakers	−	− 0.056* (0.031)
Self-evaluation of the amount of professional knowledge	−	0.013 (0.035)
*phi*^a^	5.58*** (0.53)	5.86*** (0.55)
Constant	− 0.993*** (0.343)	− 0.219 (0.415)
Observations	200	200
R^2^	0.046	0.084
Log likelihood	79.407	84.044

One asterisk refers to a *p*-value smaller than 0.1, two asterisks to a *p*-value smaller than 0.05, and three asterisks to a *p*-value smaller than 0.01; parentheses indicate the standard error of every variable.

^a^*phi* was estimated as a parameter that decided the shape of the beta-distribution for the models.

We then performed the same analyses on the Amazon review data. The results were consistent with those shown in Fig. 2 and Table 2 (see details in Section S5 of the Supplementary Information). Compared with the low professional knowledge group (i.e., with the top 25% distance to specialists), the high professional knowledge group (i.e., with the bottom 25% distance to the specialists) had a significantly higher average area ratio in their pictures (the average area ratios were 0.62 and 0.6 in the high and low professional knowledge groups, respectively; *t* = 1.67, *p* value = 0.02; *r* = 0.03); as per beta-regression, the distance to the specialists showed a significantly negative effect on the area ratio (*coefficient* =  − 0.961, *p* value < 0.01). In addition to these analyses, we examined the *causality* *relation* between participants’ amounts of professional knowledge and their attention deployment patterns based on an additional analysis (see details in Section S5 of the Supplementary Information). The results of this analysis supported our research hypothesis stating that participants’ large (small) amounts of professional knowledge *caused* their focused (divided) attention deployment patterns.

To summarise, the above results suggest that participants with more professional knowledge about the target product gave their concentrated attention to it. Contrarily, participants with less professional knowledge about the target product gave their divided attention. Moreover, since our two datasets came from Japanese and American participants, these results were consistent in the cultural backgrounds of both these countries.

### Impacts of attention deployment patterns on creative performances

**Figure 3:**
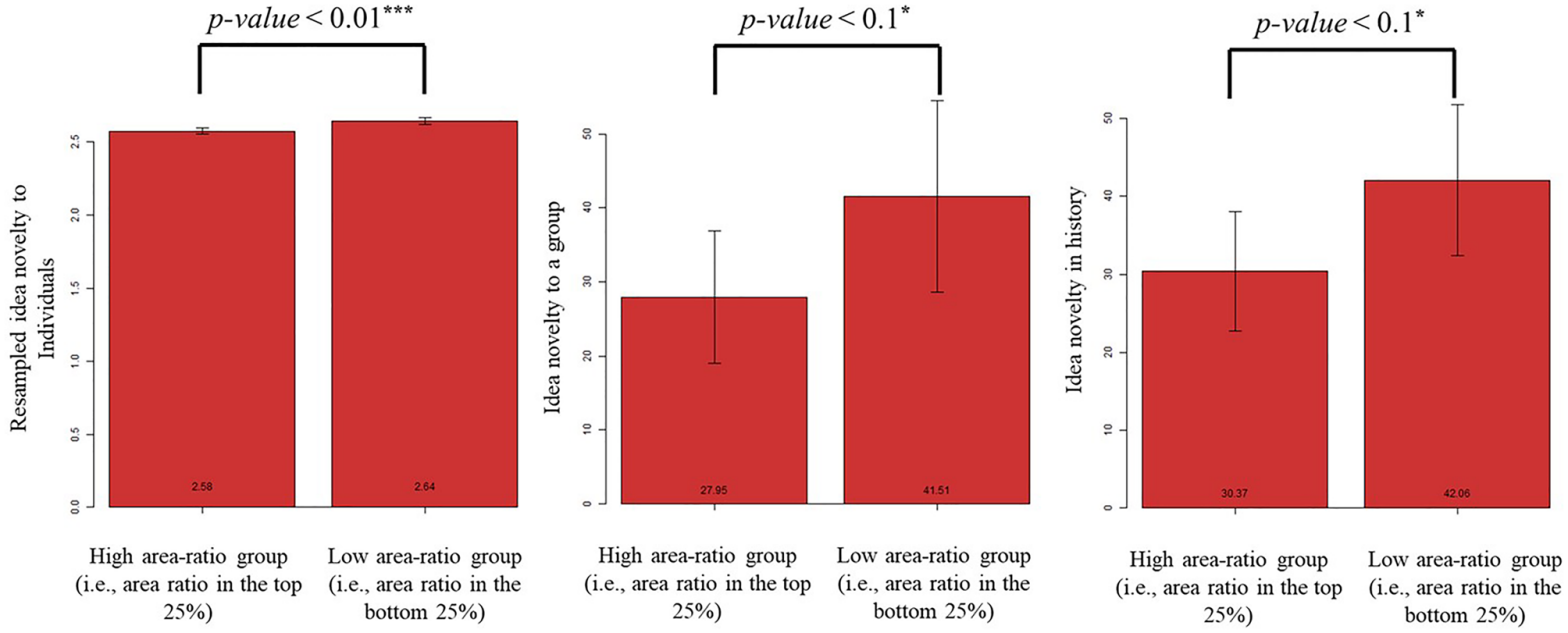
Illustration of different creative performances of the high and low area-ratio groups in the survey data. The left panel shows the results based on the resampled idea novelty to individuals; the middle panel shows the results based on the idea novelty to a group; the right panel shows the results based on the idea novelty in history. The left bar of the bar chart in all panels indicates the average value of the metric in the high area-ratio group (i.e., participants with the top 25% area ratio) while the right bar, that of the low area-ratio group (i.e., participants with the bottom 25% area ratio).

We examined the impact of different attention deployment patterns on creative performances. Fig. 3 shows the average creative performance of participants with the top 25% area ratios (i.e., the high area-ratio group) and that of those with the bottom 25% area ratios (i.e., the low area-ratio group). We found that compared with the high area ratio group, the low area-ratio group had significantly or marginally significantly higher average creative performances under all metrics: the idea novelty to individuals (high area-ratio group: 2.57; low area-ratio group: 2.64; *t* =  − 12.26, *p* value < 0.01; *r* = 0.77), idea novelty to a group (high area-ratio group: 27.95; low area-ratio group: 41.51; *t* =  − 1.73, *p* value = 0.08; *r* = 0.17), and idea novelty in history (high area-ratio group: 30.37; low area-ratio group: 42.06; *t* =  − 1.90, *p* value = 0.06; *r* = 0.19). Since the idea novelty to individuals’ skewed distribution could lead to unreliable results in the statistical test^38^, we implemented bootstrap resampling (see details in Section S6.1 of the Supplementary Information) to obtain robust results in the statistical test^39^.

Moreover, we employed regression models to examine the impact of different attention deployment patterns on creative performance. Since previous research^8,11^ has found that professional knowledge and the use of target products also affected creative performance, participants’ knowledge test scores and their basic information on speaker usage were controlled in the regression models. As explained in the above sections, the basic information on speaker usage included four variables: (1) number of speaker holdings, (2) frequency of speaker usage, (3) self-evaluation of the particularities of speakers, and (4) self-evaluation of the amount of professional knowledge about speakers. Along with these variables, participants’ gender and age were controlled. As the correlations among the independent and control variables were high (see the correlations in Fig. S1 in the Supplementary Information), we used the LASSO regression to build our models to prevent collinearity^40^. In LASSO regression, variables that do not have significant effects on the dependent variable are given zero coefficients (i.e., the coefficients of these variables will be set to zero); only variables that significantly affect the dependent variable have non-zero coefficients. Therefore, the LASSO regression is effective in finding the smallest model and preventing collinearity among variables^40^. The results of the LASSO regressions are shown in Table 3. The lambda in Table 3 is the penalty parameter of the LASSO regression. As lambda increases, the model becomes stricter towards unnecessary variables; that is, more variables will be removed from the model (i.e., the coefficients will be set to zero). The best lambdas reported in Table 3 were estimated using the AIC^40^. In all three models, the area ratio had non-zero but negative coefficients on participants’ creative performance (in Model 4 using the idea novelty to individuals as the dependent variable: *coefficient* =  − 1.17; in Model 5, using the idea novelty to a group as the dependent variable: *coefficient* =  − 17.06; in Model 6, using the idea novelty in history as the dependent variable: *coefficient* =  − 13.67). These results indicated that the target product’s area ratio in the participants’ pictures had significantly negative effects on creative performance.

**Table 3:** Regression results for creative performance in the survey data.

Dependent variable	Idea novelty to individuals	Idea novelty to a group	Idea novelty in history
	Model 3	Model 4	Model 5
Area ratio	−1.168	−17.064	−13.667
Test score	−	1.057	0.972
Gender	−	2.502	1.967
Age	−	−	−
Number of speaker holdings	−	−	−
Frequency of speaker usage	−	−	−
Self-evaluation of the particularities of speakers	−	−	−
Self-evaluation of the amount of professional knowledge about speakers	−0.013	−3.369	−3.435
Constant	0.209	42.334	46.455
Lambda^a^	0.079	1.585	1.259
Observations	200	200	200

The − indicates that the variable was eliminated (i.e., had a coefficient of zero) in the LASSO regression.

^a^The best lambda value is reported, which is estimated by the AIC of the models.

Based on the Amazon review data, we obtained the same results as shown in Fig. 3 and Table 3 (see details in Sect. 6.2 of the Supplementary Information). Compared with the top area-ratio group (participants with the top 25% area ratios), the bottom area-ratio group (those with the bottom 25% area ratios) had a significantly higher average review novelty to a group (the top area-ratio group: 5.64; the bottom area-ratio group: 6.98; *t* =  − 12.22, *p*-value < 0.01; *r* = 0.24) and in history (the top area-ratio group: 1.50; the bottom area-ratio group: 2.47; *t* =  − 7.57, *p-*value < 0.01; *r* = 0.15). In the regression models (OLS), the area ratio had a significantly negative effect on participants’ creative performance (in the model using the review novelty to a group as the dependent variable: *coefficient *=  − 1.707; *p-*value < 0.01; in the model using the review novelty in history as the dependent variable: *coefficient* =  − 1.308; *p-*value < 0.01).

These results indicated that when participants used their concentrated attention, they had a lower creative performance; contrarily, when participants used their divided attention, they had a higher creative performance.

### Path analyses: impact of amount of professional knowledge on creative performance through attention deployment patterns

**Figure 4:**
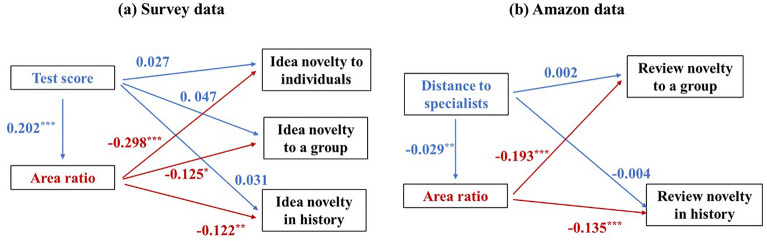
Illustration of the impact of the amount of professional knowledge on creative performance through the attention deployment pattern. The left panel shows the results based on the survey data and the right panel, based on the Amazon data. The rectangles represent the metrics of the amount of professional knowledge (in blue), attention deployment pattern (in red), and creative performance (in black). The blue lines show the impacts of the amount of professional knowledge on other variables (i.e., attention deployment pattern and creative performance). The red lines show the impacts of the attention deployment pattern on other variables (i.e., creative performance). The numbers around the lines represent the coefficient of structural equation models. The asterisks represent the sizes of the *p-values*; One asterisk refers to a *p*-value smaller than 0.1, two asterisks to a *p*-value smaller than 0.05, and three asterisks to a *p*-value smaller than 0.01. Parentheses indicate the standard error of each variable.

Finally, to combine the aforementioned results, we investigated how participants’ amount of professional knowledge affected their creative performance through attention deployment patterns. Based on previous research^41^, we conducted *path* *analyses* using the *structural* *equation* *model*. This model consisted of two parts^41^: the first part examined the relation between the amount of professional knowledge and the attention deployment pattern, and the second part examined the relation between the amount of professional knowledge and creative performance when controlling the attention deployment pattern. Fig. 4a illustrates the results of the path analyses based on the survey data. The results showed that the amount of professional knowledge (i.e., the test score) did not have a significant direct impact on creative performance (i.e., the three types of idea novelty). However, by affecting the attention deployment pattern (i.e., the area ratio), the amount of professional knowledge negatively impacted the creative performance (also see details in Table S6 in the Supplementary Information).

Based on the Amazon review data, we conducted the same path analyses. As Fig. 4b illustrates, the results were the same: the amount of professional knowledge (i.e. the distance to specialists) did not have a significant direct impact on creative performance (i.e., the two types of review novelty). However, by affecting the attention deployment pattern (i.e., the area ratio), the amount of professional knowledge negatively impacted the creative performance (see details in Table S7 in the Supplementary Information).

Therefore, to summarise, participants with more (less) professional knowledge used their concentrated (divided) attention that affected their low (high) creative performance.

## Discussion

In this study, we empirically showed that people with less professional knowledge give their divided attention, which positively affects their creative performances. However, people with more professional knowledge use their concentrated attention, which negatively affects their creative performances***.*** Our research contributes to the academic studies in the corresponding fields and the creative practices in the real world.

Academically, previous research^12–20,42–44^ on attention deployment patterns has found that several factors, such as the environment of information processing and positive affect, impact participants’ attention deployment patterns. However, to the best of our knowledge, the relation between the amount of professional knowledge and attention deployment patterns remained unexplored. In this study, we examined this relation. A large amount of professional knowledge leads to a focused attention deployment pattern; contrarily, a small amount leads to a divided attention deployment pattern. Based on these results, we also uncovered the cognitive mechanism behind the phenomenon wherein people with less professional knowledge can achieve higher creative performances than those with more professional knowledge. It is because people with less professional knowledge use their divided attention more during information processing. Finally, in terms of methodology, this study employed computer-vision and natural-language-processing methods in computer science to develop the metrics of the attention deployment patterns and creative performances. These metrics can deepen the discussions on cognitive differences and creative performances in future research.

In practical terms, first, the results of this research expanded the application range of crowd-sourcing innovation, which is a new way of utilising ideas from a group of people outside the organisation to achieve innovations^9^. Previous research^9–11^ has found that crowd-sourcing innovation is an effective way to achieve innovations involving usage experiences (e.g., add a new function to speakers). Therefore, our results implied that crowd-sourcing innovation is also effective when the innovations require a large amount of professional knowledge (e.g., the development of new materials for food packages; see details in https://innocentive.wazoku.com/#/challenge/3ebd1b5a7cc34ed1a299cc34e09a7736?searchIndex=2) ^45^. Specifically, even when faced with professional issues, amateurs (i.e., people with less professional knowledge) in a crowd-sourcing innovation group would also be able to generate more and better ideas than the R&D teams in companies^45^. Second, our results can contribute to organisation design in the real world. When managers attempt to organise a team for creative activities (e.g., the R&D team), our results implied that a group of amateurs, instead of a group of specialists (i.e., people with significant professional knowledge), could achieve a higher performance^46,47^. Third, our research pointed out the problem of specialists during creative activities: they tend to focus their attention in an extremely concentrated way that hinders their ability to find new information combinations. Hence, by helping specialists to divide their attention across a wider scope, their creative performances would improve.

There are three main limitations of this research. First, the discussions on user innovation focused only on participants’ *amount* of professional knowledge. However, the *diversity* (i.e., combination) of their professional knowledge also largely affects their creative performances in user innovation^9–11^. Especially, previous research^48^ has found that participants with multidisciplinary knowledge can also get high performances in user innovation. Therefore, how the diversity (i.e., combination) of professional knowledge affects one’s creative performances during user innovation should be discussed in future research.

Second, following previous studies, this research only discussed how focused and divided attention deployment patterns affect creative performances. However, as explained in the Introduction, attention deployment includes other aspects (e.g., the sustainability aspect and aspects involving covert vs. overt attention). The issue of how these other aspects of attention deployment affect creative performance should be discussed in future research. Especially, based on a more comprehensive dataset, which consistently records both participants’ attention deployment patterns and their response times during creative activities, future research should address the relation between the sustainability of one’s attention deployment and their creative performance. In addition to sustainability, because of data availability, this research did not distinguish participants’ attention deployment as an overt one (i.e., deployed her/his attention consciously) or a covert one (i.e., deployed attention unconsciously). In this regard, future research should investigate an interesting issue of how covert and overt attention deployments differently affect participants’ creative performances in creative activities.

Third, in this research, we developed several new metrics to measure participants’ amounts of professional knowledge, attention deployment patterns, and creative performances based on computer science methods. To examine the robustness of the results based on these new metrics, this research showed the consistency among the results across two different datasets (i.e., the survey data and Amazon review data). However, there are still many other potential ways to examine the robustness of our results, especially those based on the Amazon review dataset. For instance, previous research^49^ has found that previous job experience has a strong correlation with a participant’s amount of professional knowledge. In addition, attention deployment patterns can be reflected in people’s ways of categorisation^23,24^ (see details in Section S3.2 in the Supplementary Information). Finally, creative performance has a correlation with one’s information searching pattern (e.g., focusing on divergent or related information)^50^. Using a new dataset, including data on user profiles and their searching history on different products, future research may build different metrics based on the above studies and re-test the robustness of the results in this research.

## Materials and methods

### (1) Description of survey and Amazon datasets

In this study, we collected two datasets: a web-based survey data from 200 Japanese participants and the Amazon review data comprising 201,489 reviews of speakers from American participants.

To gather the participants in the web-based survey, we contracted with *Rakuten* *Insight* (https://member.insight.rakuten.co.jp/), which is a famous investigation company in Japan. Rakuten Insight has the largest panel in Japan consisting of more than 220,000 people. Participants in the web-based survey (age: *mean* = 49.5, s*t.dev* = 12.6; gender: *female* *prob* = 14%) were randomly selected from the Rakuten Insight’s panel under the following conditions: (1) age ranges from 20 to 60 years old. (2) owns at least one speaker at home; and (3) the speaker(s) were bought based on the participant’s will. All participants provided informed consent prior to study enrolment. The experimental protocol was approved by the University of Tokyo Research Ethics Committee and conducted in accordance with the latest version of the Declaration of Helsinki. Every participant in the survey was required to (1) participate in a knowledge test (see details in Section S2 of the Supplementary Information) concerning speakers, (2) submit a picture to introduce their speakers, and (3) submit an idea of future speakers (i.e., collected through the question: ‘Considering the development of speakers 5 years from now, please submit an idea of the new functions of speakers in 2025’). We also gathered other control variables that were used to build the regression models. The details of these variables can be found in Section S2 of the Supplementary Information.

We gathered the Amazon review data from the well-known online shopping website, Amazon.com (U.S.). Using the product-filtering function in Amazon (U.S.), we gathered 201,489 reviews of all 424 products sold under the ‘speaker’ category (published up to 1 June 2019). Additionally, in a part of the reviews (i.e., 4857 reviews), the pictures of the target products (i.e., speakers) were uploaded. All 9257 pictures were also gathered.

### (2) Computation of the distance to the known specialists based on the Amazon review data

In the Amazon review data, based on previous research^31–33^, we measured the amount of professional knowledge of Amazon participants using the review texts. First, we gathered 645 specialists’ reviews of speakers (i.e., the known specialists’ reviews; published up to 1 June 2019) from two well-known professional technical blogs: CNET (https://www.cnet.com/) and Digital Trends (https://www.digitaltrends.com/). These specialists’ reviews were uploaded by the professional reviewers of speakers hired by the above two technical blogs. Next, using the document-embedding model Doc2Vec^34^, we computed every Amazon review text’s cosine distance to the known specialists’ reviews (i.e., the distance to the specialists). Doc2Vec uses a neural network to obtain the vector representation of the target text. Previous studies^34,51–53^ have found that a smaller cosine distance between two vectors represents higher similarity between two texts. Therefore, the Amazon participants who had a smaller distance to the specialists were considered as those with more professional knowledge, whereas those who had a larger distance to the specialists were those with less professional knowledge.

### (3) Computation of area ratio

**Figure 5:**
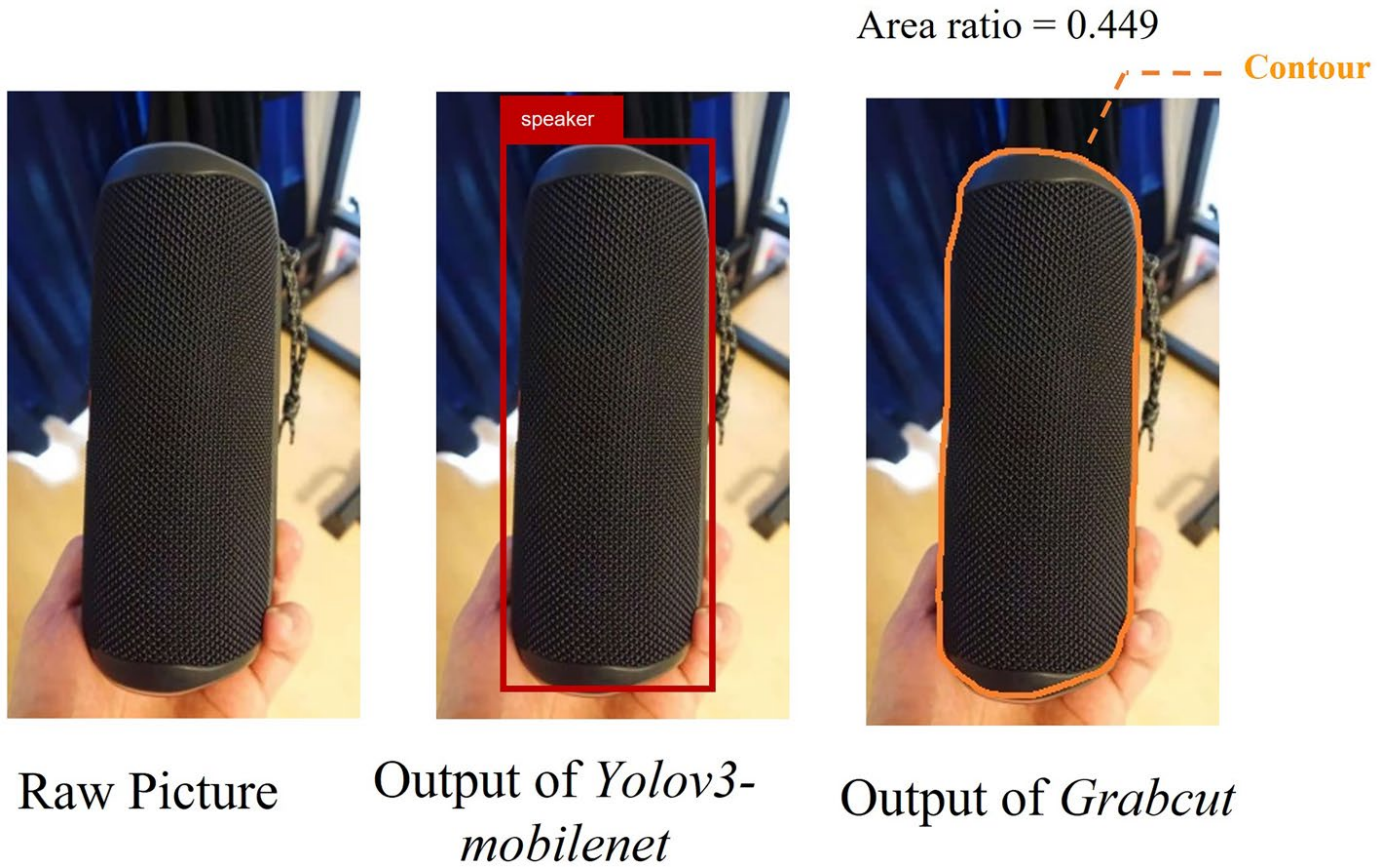
Illustration of the output of Yolov3-Mobilenet and Grabcut. The left panel shows the input (i.e., raw picture) of our models; the middle panel shows the output of Yolov3-mobilenet and the right panel shows that of Grabcut. Yolov3-mobilenet detected the approximate location (red rectangle) of the speaker in the raw picture. Grabcut detected the pixel-level contour (in orange) of the speaker based on the Yolov3-mobilenet output. The area ratio can then be easily computed from the output of Grabcut. The area ratio in this picture was 0.449. The picture used in this figure wa**s** taken by the first author. Therefore, thi**s** was only used for illustration but not a real example in our two datasets.

In our two datasets, we measured participants’ attention deployment patterns based on the area ratio in their pictures. To compute the ratio, we combined two well-known computer-vision models: Yolov3-Mobilenet and Grabcut^25–27^. The former, which is based on a neural network, was used to recognise the approximate location of the speaker in pictures. The latter, which is based on a classification algorithm, was used to recognise the pixel-level contour of the speaker in pictures. To train the Yolov3-Mobilenet to recognise speakers, we labelled, by hand, the approximate locations (as shown in the middle panel of Fig. 5) of the speakers in 400 randomly selected pictures from our two datasets and used them as training data. As shown in Fig. 5, by combining Yolov3-mobilenet and Grabcut, we could automatically identify the pixel-level contours of the speakers in our picture data. Based on their contours, the area ratio could be easily calculated.

To verify the model-generated area ratios, we randomly selected 100 pictures from our datasets and computed the area ratios by hand (see details in Section S3.1 of the Supplementary Information). We found that the correlation between the model- and human-generated area ratios was as high as 0.80 (*p*-value < 0.01).

### (4) Computation of idea novelty to individuals

To measure the idea novelty of individuals, we asked seven experts of speakers to evaluate participants’ ideas. Among these seven experts, four (i.e., experts No. 1 to 4) belonged to the R&D team of a world-famous audio device manufacturer in Japan; two experts (i.e., experts No. 5 and 6) were researchers in the fields of information engineering and cognitive science at two different universities in Japan; and one expert (i.e., expert No. 7) was from a very famous consulting company in Japan. A 5-point scale question was used for the evaluation, from 1 = *very* *low* *idea* *novelty* to 5 = *very* *high* *idea* *novelty*^28^. During the evaluation, the experts were asked to evaluate the idea novelty by comprehensively considering three factors^28–30^: (1) the uniqueness of the expression of the idea, (2) the difficulty to generate the idea, and (3) the quality of the idea. To justify the robustness of these evaluations, we investigated the correlations among the seven experts’ evaluation scores. We found significantly positive correlations (ranging from 0.26 to 0.63; see the specific correlations in S8 in the Supplementary Information) among the experts’ evaluation scores. These results indicate that for the same idea, the seven experts provided consistent evaluations on its novelty.

Based on these evaluations, for every idea, we used the median of the seven evaluation scores as its idea novelty to individuals^28–30^.

### (5) Computation of idea novelty to a group and review novelty to a group

In the survey data, to measure the idea novelty to a group, we computed the *Tf-idf* (i.e., term frequency–inverse document frequency) of every idea. *Tf-idf* is an effective indicator used to assess the rare occurrence (i.e., novelty) of a word or text in a set of texts^54^. For every idea, we first computed the *Tf-idf*s of all words: Given the ideas collection *D,* word *w*, and focused idea *d*, the *Tf-idf* of the word *w* in idea *d* is calculated as follows^54^:

$$Tfid{f}_{w}={p}_{w,d}\times {\mathrm log}({\frac{\left|D\right|}{ f_{w,D}}})$$

where ${p}_{w,d}$ is equal to the number of times *w* appeared in *d* divided by the number of all words’ appearances in *d*; $\left|D\right|$ is the number of all ideas; and ${f}_{w, D}$ equals the number of ideas in which *w* appeared. One word’s *Tf-idf* indicates how novel the word is when compared with all words in other ideas; therefore, one idea’s *Tf-idf* equals the sum of all words’ *Tf-idfs* for this idea^53–55^:

$$Tfid{f}_{d}= {\sum }_{w\in d}Tfid{f}_{w} = {\sum }_{w\in d}{p}_{w,d}\times {\mathrm log}({\frac{\left|D\right|}{f_{w,D}}})$$

$Tfid{f}_{d}$ indicates how novel an idea *d* is compared with all other ideas. It indicates the idea novelty to the group of idea *d*^55^.

Using the same methods as above (i.e., the *Tf-idf* of the review), we constructed the review novelty to a group in the Amazon review data. The details of our methods can be found in Section S4.2 of the Supplementary Information.

### (6) Computation of idea novelty in history and review novelty in history

In the survey data, to measure the idea novelty in history, we first needed a dataset that generally included speaker-related information. Based on previous research^56^, we utilised 49,818 speaker-related Wikipedia pages (see details in Section S4 of the Supplementary Information). Based on these Wikipedia pages, we computed the communication burden of every idea as an indicator of the idea’s novelty to history^57^. Especially, the communication burden addresses the rare occurrence (i.e., novelty) of a word or text when the texts belong to different categories (i.e., one idea vs. all Wikipedia pages)^57^. For every idea, we first computed the communication burdens of all words: given the Wikipedia pages collection *Q*, a word *w*, and the focused idea *d*, the communication burden of *w* was calculated as follows^57^:

$${CB}_{w}=-{p}_{w,d}\times \mathrm{log}\it{({p}_{w,Q}})$$

where ${p}_{w,d}$ is equal to the number of times *w* appeared in *d* divided by the number of all words’ appearances in *d*; ${p}_{w,Q}$ equals the number of times *w* appeared in *Q* divided by the number of all words’ appearances in *Q.* The communication burden of one word indicates how novel the word is compared with all words in the Wikipedia pages; accordingly, the communication burden of an idea equals the sum of communication burdens of all words:

$$C{B}_{d}={\sum }_{w\in d}C{B}_{w} ={\sum }_{w\in d}-{p}_{w,d}\times \mathrm{log}({\it{p}_{w,Q}})$$

$C{B}_{d}$ indicates how novel an idea *d* is compared with all Wikipedia pages. It indicates the idea novelty in history of idea *d*^57^.

Using the same methods as above (i.e., the communication burden of the review), we constructed the review novelty in history in the Amazon review data. The details of our methods can be found in Section S4.2 of the Supplementary Information.

## Code and data availability

The R-code of the analyses in and the two datasets analysed during the current study are available in the following URL: https://github.com/yoguluto/Different-attention-deploying-patterns.

## Supplementary Information

Supplementary Information.

## References

[1] Kirstetter E, Eagar R, Kolk M, Roos D (2013). The creativity era—A new paradigm for business. PRism.

[2] Granovetter, M. *The**Sociology**of**Economic**Life.* (Routledge, 2018).

[3] Phelps C, Heidl R, Wadhwa A (2012). Knowledge, networks, and knowledge networks: A review and research agenda. J. Manag..

[4] Thornhill S (2006). Knowledge, innovation and firm performance in high-and low-technology regimes. J. Bus. Ventur..

[5] Williamson OE (1988). Technology and transaction cost economics: A reply. J. Econ. Behav. Organ..

[6] Nelson, R. R. *An**Evolutionary**Theory**of**Economic**Change* (Harvard University Press, 2009).

[7] Fleming L, Sorenson O (2004). Science as a map in technological search. Strateg. Manag. J..

[8] Von Hippel E (1986). Lead users: A source of novel product concepts. Manag. Sci..

[9] Gustafsson A, Kristensson P, Witell L (2012). Customer co-creation in service innovation: A matter of communication?. J. Serv. Manag..

[10] Huang Y, Vir Singh P, Srinivasan K (2014). Crowdsourcing new product ideas under consumer learning. Manag. Sci..

[11] Hallikainen H, Alamäki A, Laukkanen T (2019). Lead users of business mobile services. Int. J. Inform. Manag..

[12] Kasof J (1997). Creativity and breadth of attention. Creat. Res. J..

[13] Memmert D (2007). Can creativity be improved by an attention-broadening training program? An exploratory study focusing on team sports. Creat. Res. J..

[14] Memmert D (2006). Developing creative thinking in a gifted sport enrichment program and the crucial role of attention processes. High Abil. Stud..

[15] Batey M, Furnham A (2006). Creativity, intelligence, and personality: A critical review of the scattered literature. Genet. Soc. Gen. Psychol. Monogr..

[16] Agnoli S, Franchin L, Rubaltelli E, Corazza GE (2015). An eye-tracking analysis of irrelevance processing as moderator of openness and creative performance. Creat. Res. J..

[17] Chen J, Wang RQ, Lin Z, Guo X (2018). Measuring the cognitive loads of construction safety sign designs during selective and sustained attention. Saf. Sci..

[18] Vanderhaegen F, Wolff M, Mollard R (2020). Non-conscious errors in the control of dynamic events synchronized with heartbeats: a new challenge for human reliability study. Saf. Sci..

[19] Masuda T, Gonzalez R, Kwan L, Nisbett RE (2008). Culture and aesthetic preference: Comparing the attention to context of East Asians and Americans. Pers. Soc. Psychol. Bull..

[20] Jennings AE, Chiles WD (1977). An investigation of time-sharing ability as a factor in complex performance. Hum. Factors.

[21] Wickens CD, Mountford SJ, Schreiner W (1981). Multiple resources, task-hemispheric integrity, and individual differences in time-sharing. Hum. Factors.

[22] Lansman M, Poltrock SE, Hunt E (1983). Individual differences in the ability to focus and divide attention. Intelligence.

[23] Nisbett RE, Masuda T (2003). Culture and point of view. Proc. Natl. Acad. Sci. U.S.A..

[24] Nisbett RE, Miyamoto Y (2005). The influence of culture: Holistic versus analytic perception. Trends Cogn. Sci..

[25] Tang, M., Gorelick, L., Veksler, O. & Boykov, Y. Grabcut in one cut. in *Proceedings**of**the**2013**IEEE**International**Conference**on**Computer**Vision* 1769–1776 (IEEE, 2013).

[26] Huang R, Gu J, Sun X, Hou Y, Uddin S (2019). A rapid recognition method for electronic components based on the improved YOLO-V3 network. Electronics.

[27] Wang, J., Xiao, W. & Ni, T. Efficient object detection method based on improved YOLOv3 network for remote sensing images. in *Proceedings**of**the**3rd**International**Conference**on**Artificial**Intelligence**and**Big**Data* 242–246 (IEEE, 2020).

[28] Dean DL, Hender J, Rodgers T, Santanen E (2006). Identifying good ideas: Constructs and scales for idea evaluation. J. Assoc. Inf. Syst..

[29] Kudrowitz BM, Wallace D (2013). Assessing the quality of ideas from prolific, early-stage product ideation. J. Eng. Des..

[30] Silvia PJ (2008). Assessing creativity with divergent thinking tasks: Exploring the reliability and validity of new subjective scoring methods. Psychol. Aesthet. Creat. Arts..

[31] Johnson GM, Kulpa A (2007). Dimensions of online behavior: Toward a user typology. Cyberpsychol. Behav..

[32] Brandtzæg PB (2010). Towards a unified media-user typology (MUT): A meta-analysis and review of the research literature on media-user typologies. Comput. Hum. Behav..

[33] Brandtzæg PB, Heim J, Karahasanović A (2011). Understanding the new digital divide—A typology of internet users in Europe. Int. J. Hum-Comput. St..

[34] Le, Q. & Mikolov, T. Distributed representations of sentences and documents. in *Proceedings**of**the**31st**International**Conference**on**Machine**Learning* (eds. Xing, E. P. & Jebara, T.) 1188–1196 (JMR, 2014).

[35] Ferrari S, Cribari-Neto F (2004). Beta regression for modelling ratios and proportions. J. Appl. Stat..

[36] Abouserie R, Moss D, Barasi S (1992). Cognitive style, gender, attitude toward computer-assisted learning and academic achievement. Educ. Stud..

[37] Riding RJ, Grimley M, Dahraei H, Banner G (2003). Cognitive style, working memory and learning behaviour and attainment in school subjects. Br. J. Educ. Psychol..

[38] Fay MP, Proschan MA (2010). Wilcoxon-Mann-Whitney or t-test? On assumptions for hypothesis tests and multiple interpretations of decision rules. Stat. Surv..

[39] Lahiri, S. N. *Resampling**Methods**for**Dependent**Data* (Springer, 2013).

[40] Tibshirani R (2011). Regression shrinkage and selection via the LASSO: A retrospective. J. R. Stat. Soc. Ser. B Stat. Methodol..

[41] Zhao X, Lynch JG, Chen Q (2010). Reconsidering Baron and Kenny: Myths and truths about mediation analysis. J. Consum. Res..

[42] Müller BC, Gerasimova A, Ritter SM (2016). Concentrative meditation influences creativity by increasing cognitive flexibility. Psychol. Aesthet. Creat. Arts.

[43] Isen AM (2001). An influence of positive affect on decision making in complex situations: Theoretical issues with practical implications. J. Consum. Psychol..

[44] Kaplan S, Tripsas M (2008). Thinking about technology: Applying a cognitive lens to technical change. Res. Policy.

[45] Davis K (2015). InnoCentive.com collaboration case study. J. Manag. Policies Pract..

[46] Lilien GL, Morrison PD, Searls K, Sonnack M, Hippel E (2002). Performance assessment of the lead user idea-generation process for new product development. Manag. Sci..

[47] Fuchs C, Schreier M (2011). Customer empowerment in new product development. J. Prod. Innov. Manag..

[48] DiCarlo, L., McGowan, H. & Rottenberg, S. *Handbook**of**Anthropology**in**Business* (Routledge, 2016).

[49] Schmidt FL, Hunter JE, Outerbridge AN (1986). Impact of job experience and ability on job knowledge, work sample performance, and supervisory ratings of job performance. J. Appl. Psychol..

[50] Dubitzky, W., Kötter, T., Schmidt, O. & Berthold, M. R. Towards creative information exploration based on Koestler’s concept of bisociation. in *Bisociative**Knowledge**Discovery* (ed. Berthold, M. R.) 11–32 (Springer, 2012).

[51] Lau, J. H. & Baldwin, T. *An**Empirical**Evaluation**of**doc2vec**with**Practical**Insights**into**Document**Embedding**Generation*. Preprint: https://arxiv.org/abs/1607.05368 (2016).

[52] Trieu, L. Q., Tran, H. Q. & Tran, M.-T. News classification from social media using twitter-based doc2vec model and automatic query expansion. in *Proceedings**of**the**8th**International**Symposium**on**Information**and**Communication**Technology* 460–467 (ACM, 2017).

[53] Kim D, Seo D, Cho S, Kang P (2019). Multi-co-training for document classification using various document representations: TF-IDF, LDA, and Doc2Vec. Inf. Sci..

[54] Ramos, J. Using tf-idf to determine word relevance in document queries. in *Proceedings**of**the**12th**International**Conference**on**Machine**Learning*, (eds. Fawcett, T. & Mishra, N.) 133–142 (AAAI, 2003).

[55] Shi F, Teplitskiy M, Duede E, Evans JA (2019). The wisdom of polarized crowds. Nat. Hum. Behav..

[56] Burke, P. *A**Social**History**of**Knowledge**II:**From**the**Encyclopaedia**to**Wikipedia* (Polity Press, 2012).

[57] Vilhena DA (2014). Finding cultural holes: How structure and culture diverge in networks of scholarly communication. Sociol. Sci..

