# Supplementary material for: More knowledge causes a focused attention deployment pattern leading to lower creative performances
Source: Sci Rep. 2021 Sep 14;11:18062. doi: 10.1038/s41598-021-97215-5 (PMC8440603; doi:10.1038/s41598-021-97215-5)
Supplement: Supplementary file 1 — Supplementary Information. [file 41598_2021_97215_MOESM1_ESM.pdf]

Supplementary Information for

More knowledge causes a focused attention deployment pattern leading to lower creative performances

Authors: Kunhao Yang[1]*, Itsuki Fujisaki[1,2], Kazuhiro Ueda[1]*

[1] Graduate School of Arts and Sciences, The University of Tokyo, Tokyo, Japan (〒153-8902).

[2] Research Fellowship for Young Scientists (DC2), Japan Society for the Promotion of Science (JSPS), Tokyo, Japan (〒102-0083).

* Kunhao Yang, Kazuhiro Ueda

Email: yangkunhao@g.ecc.u-tokyo.ac.jp; ueda@gregorio.c.u-tokyo.ac.jp

This PDF file includes:

Supplementary text

Figures S1 to S7

Tables S1 to S7

SI References

Supplementary Information Text

S1. Statistical information of all variables in the regression models 1–5

Table S1 shows the statistical information on all variables that were used to build regression models 1–5 in the study. Fig. S1 presents the distributions of all these variables and the correlations among them.

-----Fig. S1 about here-----

-----Table S1 about here-----

S2. Details of the knowledge test and data collections of control variables in the survey data

In the survey data, we measured participants' amount of knowledge based on their scores on a speaker knowledge test. The full score of the knowledge test was 20. All 11 questions in the test were extracted from five well-known certification exams of speakers and audio devices in Japan. They were the: 1) Technical Skills Test of Stage Effect Adjustment (http://www.javada.or.jp/), 2) Audio and Radio Skill Certification (https://ar-kentei.com/), 3) Post-Production Technician Certification Exam (http://www.jppanet.or.jp/nintei/nintei-9.html), 4) Sound Recording Technology Certification Exam (https://www.japrs.or.jp/exam/), and 5) Sound Technician Performance Test (https://www.seas-jp.org/%E9%9F%B3%E9%9F%BF%E5%AE%B6%E6%8A%80%E8%83%BD%E8%AA%8D%E5%AE%9A%E8%AC%9B%E5%BA%A7/).

In the knowledge test, participants with higher scores were considered to have more professional knowledge of speakers; by contrast, participants with lower scores were considered to have less professional knowledge of speakers.

Moreover, to build the regression models in the study, we gathered participants' gender and age and the basic information on speaker usage in the online survey and used them as control variables. The basic information on participants' speaker usage included four specific variables: 1) number of speaker holdings (collected by the question: 'How many speakers do you own? Please count only the speakers that you completely own.'), 2) frequency of speaker usage (assessed through the following question scored on a 7-point scale, ranging from 1 (*several times every day*) to 7 (*less than once per month*): 'On average, how frequently do you use your speakers?); 3) self-evaluation of the particularities of speakers (collected through the following question scored on a 7-point scale, ranging from 1 (*not at all particular*) to 7 (*very particular*): 'To what extent do you think you are particular about speakers?'), and 4) self-evaluation of the amount of professional knowledge about speakers (collected through the following question scored on a 7-point scale, ranging from 1 (*nearly none*) to 7 (*very much*): 'To what extent do you think you have professional knowledge about speakers?').

Previous research[1,2] has shown that along with control variables, the number of idea submissions should be controlled during the measurement of creative performances. Therefore, in this research, every participant was asked to submit only one idea during the survey. In other words, the number of idea submissions was controlled during the survey stage (i.e., the data collection stage) in this research.

S3.1. Verification of model-generated area ratios

To compute the area ratio in every picture accurately, we combined two effective methods in the computer-vision domain: Yolov3-Mobilenet and Grabcut[3-5].

To verify these model-generated area ratios, we randomly selected 100 pictures from our datasets. The first author and a volunteer (who did not know the purpose of and was not involved in any analysis in this research) computed the area ratios of these 100 pictures by hand coding. The correlation between these two people's human-generated area ratios was 0.901 (p-value < 0.01), and the correlation between the model- and human-generated area ratios was 0.802 (p-value < 0.01).

S3.2. Verification of the relation between area ratios and attention deployment patterns

To verify the relation between area ratios and attention-deployment patterns, we implemented an additional analysis based on the survey data. Previous studies[6-8] ascertain that participants' attention-deployment patterns have a strong correlation with their ways of categorisation. When participants deploy their concentrated attention, they focus on the *taxonomic* relations during categorisation. By contrast, when participants deploy their divided attention, they focus on the *thematic* relations during categorisation. The taxonomic relations reflect the similarity of concepts based on categories, whereas the thematic relations are based on the same scenario or

event[9]. For instance, dogs and bears are taxonomically similar because they belong to the same category (i.e., mammals); however, dogs and leads are thematically similar because they often occur in the same scenario (i.e., walking a dog).

Therefore, based on previous research[6], we measured participants' ways of categorisation using 38-word-categorisation questions in the survey data, to verify the relation between area ratios and attention deployment patterns. In every question, participants were given a target word (e.g., dogs) and two words as choices (e.g., bears and leads)[6]. They were asked to select the one that they considered more similar to the target word. In every question, one choice was taxonomically similar to the target word while the other was thematically similar. By counting the number of taxonomically similar words chosen by participants, we could directly measure to what extent they focused on the taxonomic relations during their categorisation.

Fig. S2 shows that participants with larger area ratios (i.e., who have the top 50% area ratios) have a significantly higher average number for choosing taxonomically similar words than those with smaller area ratios (i.e., who have the bottom 50% area ratios) (19.09 taxonomically similar words for the top 50% area-ratio group *vs* 14.46 words for the bottom 50% area-ratio group; $t = 2.45$; *p-value* = 0.015). Participants with the focused attention deployment pattern focused on the taxonomic relations during categorisation; this result supports the relation between area ratios and attention deployment patterns. Participants with larger area ratios have more focused attention deployment patterns, whereas those with smaller area ratios have more divided attention deployment patterns.

-----Fig. S2 about here-----

S4.1. Data collection of Wikipedia pages

To measure the idea novelty to history, we needed a dataset that generally includes speaker-related information in history. Based on previous research[10], we utilised the data from Wikipedia. Using the same methods as in previous studies[11-13], we defined the speaker-related pages by the citation relation in Wikipedia: We started from a seed-page, the page of the item of speaker in the Japanese Wikipedia (https://ja.wikipedia.org/wiki/%E3%82%B9%E3%83%94%E3%83%BC%E3%82%AB%E3%83%BC). We then collected all Wikipedia pages that were cited to explain the content on the seed-page (hereafter, referred to as the *one-path pages*). Next, we took these one-path pages as the new seed-pages and gathered pages that were cited by the one-path pages (hereafter, referred to as the *two-path pages*). We stopped at the two-path pages because previous studies[9-11] have found that information in the three-path pages is unrelated to seed-pages. Based on previous studies[9-11], these Wikipedia pages comprised a sample of the speaker-related information in history.

S4.2. Metrics of creative performances based on the Amazon review data

In the Amazon review data, we built the metrics of creative performances based on review texts. Previous studies[14-16] have shown that the foundation of novel idea generation is *a novel combination of information*. We considered that the novelty of review texts reflected the ability of Amazon participants to find novel information

combinations. Therefore, the *review novelty* was used as an indirect indicator of the creative performances of Amazon participants.

Since evaluating all 201,489 reviews is very time-consuming, previous studies[14,15] have shown that *novelty to individuals* is less important in creative performance evaluation; therefore, we only computed the *review novelty to a group* and *review novelty in history* based on the *Amazon review data*.

To build the metric of the review novelty to a group, we calculated the *Tf-idf* of every review text by comparing the words in the focused review text with those in all other review texts. Given the review collection *R*, a word *w*, and the focused review *r*, the *Tf-idf* of review *r* was calculated as follows:

$$Tfidf_r = \sum_{w \in r} Tfidf_w = \sum_{w \in r} p_{w,d} \times \log\left(\frac{|R|}{f_{w,R}}\right)$$

where $p_{w,r}$ is equal to the number of times *w* appeared in *r* divided by the number of times all words appeared in *r*; $|R|$ is the number of all reviews; and $f_{w,R}$ equals the number of reviews in which *w* appears. Since the *Tf-idf* of a word indicates how novel the word is compared with all words in other reviews, one review's *novelty to a group* (i.e., $Tfidf_r$) equals the sum of all words' *Tf-idfs* in the review[17,18].

To build the metric of the review novelty in history, we gathered speaker-related Wikipedia pages in English Wikipedia according to the same method explained in Section S4.1. We finally gathered 16,863 speaker-related English Wikipedia pages. Next, we calculated the *communication burden* of every review text by comparing the words in the focused review text with those in all Wikipedia pages. Given the Wikipedia pages collection *Q*, a word *w*, and the focused review *r*, the communication burden of *w* was calculated as follows[19]:

$$CB_r = \sum_{w \in r} CB_w = -\sum_{w \in r} p_{w,r} \times \log(p_{w,Q})$$

where $p_{w,r}$ is equal to the number of times w appeared in r divided by the number of appearances of all words in r; $p_{w,Q}$ equals the number of times w appeared in r divided by the number of times all words appeared in Q. The communication burden of one word indicates how novel the word is compared with all words in the Wikipedia pages; accordingly, a review's novelty in history (i.e., CB_d) equals the sum of communication burdens of all words in the review.

S5. Different attention deployment patterns among people with different amounts of professional knowledge based on the Amazon review data

To examine the attention deployment patterns among people with different amounts of professional knowledge based on the Amazon review data, we first used *a t*-test to compare the area ratios between participants with *distances to specialists at the bottom 25%* (i.e., the high professional knowledge group) and those with *distances to specialists at the top 25%* (i.e., the low professional knowledge group). Because the area ratios ranged from 0 to 1, before the implementation of the *t*-test, we first transformed the area ratio using the arcsine transformation (i.e., $x_{new} = arcsine(x^2)$). As shown in Fig. S3, the high professional knowledge group had a significantly larger average area ratio than the low professional knowledge group (the average area ratios were 0.62 and 0.6 in the high and low professional knowledge groups, respectively; *t* = 1.67, *p*-value = 0.02).

Using the beta-regression model, we examined the relation between the *distance to specialists* and area ratio. In this model, we added the IDs of every target product as a dummy variable that only affected the constant. Therefore, the regression model estimated different constants for different target products (i.e., since there were 181 different types of speakers in our data, the model estimated 181 different constants). In other words, in this regression model, we compared the different area ratios in the pictures of the same target product. In this way, we controlled the impact of the different target products on the area ratio. Additionally, previous studies[20,21] have found that the length of the review text is closely related to participants' gender and age. Therefore, the number of words in the review was added as a control variable to indicate the potential difference between participants' gender and age. The statistical information and correlations among all variables are shown in Table S2 and Fig. S4.

As shown in Table S3, the regression results showed that the *distance to specialists* had a significantly negative coefficient (coefficient = −0.16, p-value < 0.01) for the area ratio. These results are consistent with the other results obtained in the study. They supported the research hypothesis that the large (small) amount of professional knowledge about the target product *caused* participants' focused (divided) attention deployment patterns on the target product.

An alternative explanation for the above results is that the causal relation between professional knowledge and attention deployment pattern may be opposing. This means that participants' focused (divided) attention deployment patterns may *cause* their large (small) amounts of professional knowledge about the target product. To rule out this alternative possibility, we implemented additional analyses based on

the Amazon review data. In the additional analyses, we examined the relation between participants' amounts of professional knowledge of *speakers* (i.e., the target product) and their attention deployment patterns on *other products* (e.g., digital cameras). If different amounts of professional knowledge cause different attention deployment patterns, the professional knowledge about speakers will *only* affect the attention deployment pattern on speakers. In other words, a correlation will *not exist* between participants' amounts of professional knowledge about *speakers* and their attention deployment patterns on *other products*. By contrast, if different attention deployment patterns cause different amounts of professional knowledge, a specialist will always have a focused attention deployment pattern; in other words, a positive correlation will *exist* between participants' amounts of professional knowledge about *speakers* and their attention deployment patterns on *other products*.

In the Amazon review data, 97 participants submitted a review on speakers and uploaded pictures of other products (pictures of a digital camera: 63 participants; pictures of a computer: 22 participants; pictures of a coat for women: 12 participants). Based on the same method explained in the manuscript, the area ratios of the other products in these pictures were computed. Next, we examined the correlation between the area ratios in these pictures (i.e., the attention deployment patterns on other products) and participants' distances to specialists (i.e., the amounts of professional knowledge of speakers). According to the results, a significant correlation (*coefficient* = 0.06; *p-value* = 0.912) did not exist between participants' amounts of professional knowledge of speakers and their attention-deployment patterns on other products. Considering that the area ratios of different products (i.e.,

digital cameras, computers, and coats for women) may not be comparable, we also examined the correlation between the area ratios *only* in the pictures of a digital camera and participants' distances to specialists. The results were consistent: a significant correlation (*coefficient* = -0.003; *p-value* = 0.728) still did not exist between participants' amounts of professional knowledge of speakers and their attention deployment patterns on digital cameras.

Based on the above results, we concluded that participants' different amounts of professional knowledge *caused* their different attention deployment patterns.

-----Fig. S3 about here-----

-----Table S2 about here-----

-----Fig. S4 about here-----

-----Table S3 about here-----

S6.1. Resampling of the *idea novelty to individuals*

Every idea under evaluation received one evaluation score from the seven evaluators. We used the median of these evaluation scores to indicate the novelty of this idea to individuals[14,15]. Because the idea novelty to individuals had a skewed distribution with multiple peaks (as Fig. S5 shows), which would cause unreliable results in the statistical tests[22,23], we implemented bootstrap resampling to reshape the distributions of the idea novelty to individuals. As shown in Fig. S5, given the high area-ratio group (i.e., participants with the top 25% area ratios) and the low area-ratio group (i.e., participants with the bottom 25% area ratios) under comparison, we resampled 60% (i.e., the resampling proportion; we also used 40% and 80% in the robust test)

of the participants from the two groups and computed the means of their idea novelty to individuals in the bootstrap resampling. We repeated the resampling 100 times (i.e., the resampling times; we also used 200 times in the robust test) and, finally, obtained the resampled idea novelty to individuals of the two groups. As shown in the right panel in Fig. S5, the resampled idea novelty to individuals in both groups had an approximately normal distribution. Based on this resampled idea novelty to individuals, a statistical test could robustly reflect the difference in idea novelty between the two groups[16]. In Table S4, we show that the results in Fig. 4 in the manuscript were robust under different resampling proportions and resampling times.

-----Fig. S5 about here-----

-----Table S4 about here-----

S6.2. Impact of attention deployment patterns on creative performances based on the Amazon review data

To examine the impact of attention deployment patterns on creative performances based on the Amazon review data, we first analysed the average creative performance of participants with different area ratios. Fig. S6 shows the 1) average review novelty to a group and 2) average review novelty to history between the top area-ratio group (i.e., Amazon participants with the top 25% area ratios) and the low area-ratio group (i.e., those with the bottom 25% area ratios). The results were consistent with those in Fig. 3 in the manuscript: Compared with the high area-ratio group, the low area-ratio group had a significantly higher average review novelty to a group (in the top area ratio group: 5.64; in the bottom area-ratio group: 6.98; t =

−12.22, p-value < 0.01) and average review novelty in history (in the top area ratio group: 1.50; in the bottom area-ratio group: 2.47; $t = -7.57$, p-value < 0.01).

In regression models S2–S3, we used the 1) review novelty to a group and 2) review novelty in history as dependent variables. The independent variable was the area ratio. As explained in the manuscript, since participants' gender, age, and amount of professional knowledge affect their creative performance, we added the distance to specialists and number of words in the review as control variables. The distance to specialists indicated each participant's amount of professional knowledge. Research has shown[20,21] that the number of words in the review has a high correlation with a participant's gender and age; therefore, it was added to control the potential difference in a participant's gender and age. The statistical information on all variables in the regression models can be found in Table S2. The correlations and distributions among them are shown in Fig. S4. Since there was no significant collinearity among the independent and control variables, we used the linear regression (OLS) to build the models. The results of the regressions are shown in Table S5. We found that the area ratio had significantly negative effects on creative performance (in the model using the review novelty to a group as the dependent variable: coefficient = −1.707; p-value < 0.01; in the model using the review novelty in history as the dependent variable: coefficient = −1.308; p-value < 0.01). The results were consistent with the regression results in the manuscript. They indicated that when participants deployed their concentrated attention on the target product, they had a lower creative performance. By contrast, when participants deployed their divided attention across the environment, they had a higher creative performance.

-----Fig. S6 about here-----

-----Table S5 about here-----

S7. The impact of the amount of professional knowledge on creative performances through attention deployment patterns

Finally, to examine the research hypothesis, we investigated how participants' amount of professional knowledge affected creative performances through their attention deployment patterns. Based on previous research[24], we conducted *path analyses* using structural equation models. The results are presented in Table S6 (based on the survey data) and Table S7 (based on the Amazon review data). Results showed that the amount of professional knowledge did not have a significant direct impact on creative performance. However, by affecting the attention deployment pattern, the amount of professional knowledge negatively impacted creative performance.

Therefore, the above results indicate that participants with more (less) professional knowledge deployed their concentrated (divided) attention that led to their lower (higher) creative performance.

-----Table S6 about here-----

-----Table S7 about here-----

S8. The consistency of evaluations among experts

To measure the idea novelty of individuals, we asked seven experts of speakers to evaluate participants' ideas. To justify the robustness of these evaluations, we

investigated the correlations among the seven experts' evaluation scores. As Fig. S7 shows, significantly positive correlations, ranging from 0.26 to 0.63, were found among the experts' evaluation scores. These results indicate that for the same idea, the seven experts provided consistent evaluations on its novelty.

Figures and Tables

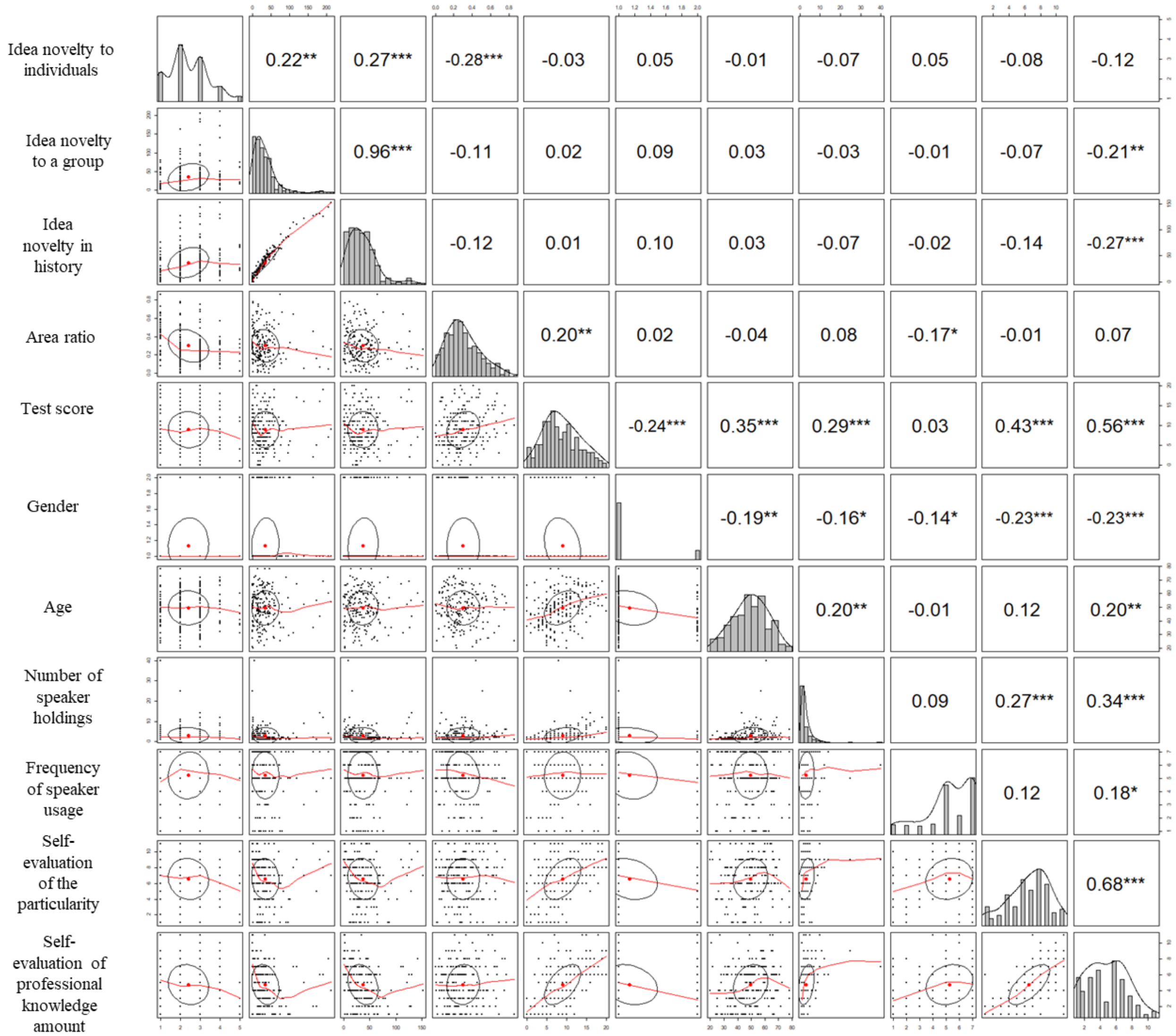

Fig. S1. Distribution, scatter plots, and correlations of all variables in regression models 1–5. The names of the variables are all to the left of the figure. In this figure, the diagonal shows the histograms, with the density curve of every variable. Graphs in the lower triangle show the scatter plots between each pair of variables. The red line shows the relation between these two variables predicted by the linear regression (OLS). The ellipses show the correlation ellipses, with the centre of each as a red point. In the upper triangle, the correlations between each pair of variables are shown. One asterisk refers to a *p*-value smaller than 0.1, two asterisks to a *p*-value smaller than 0.05, and three asterisks to a *p*-value smaller than 0.01.

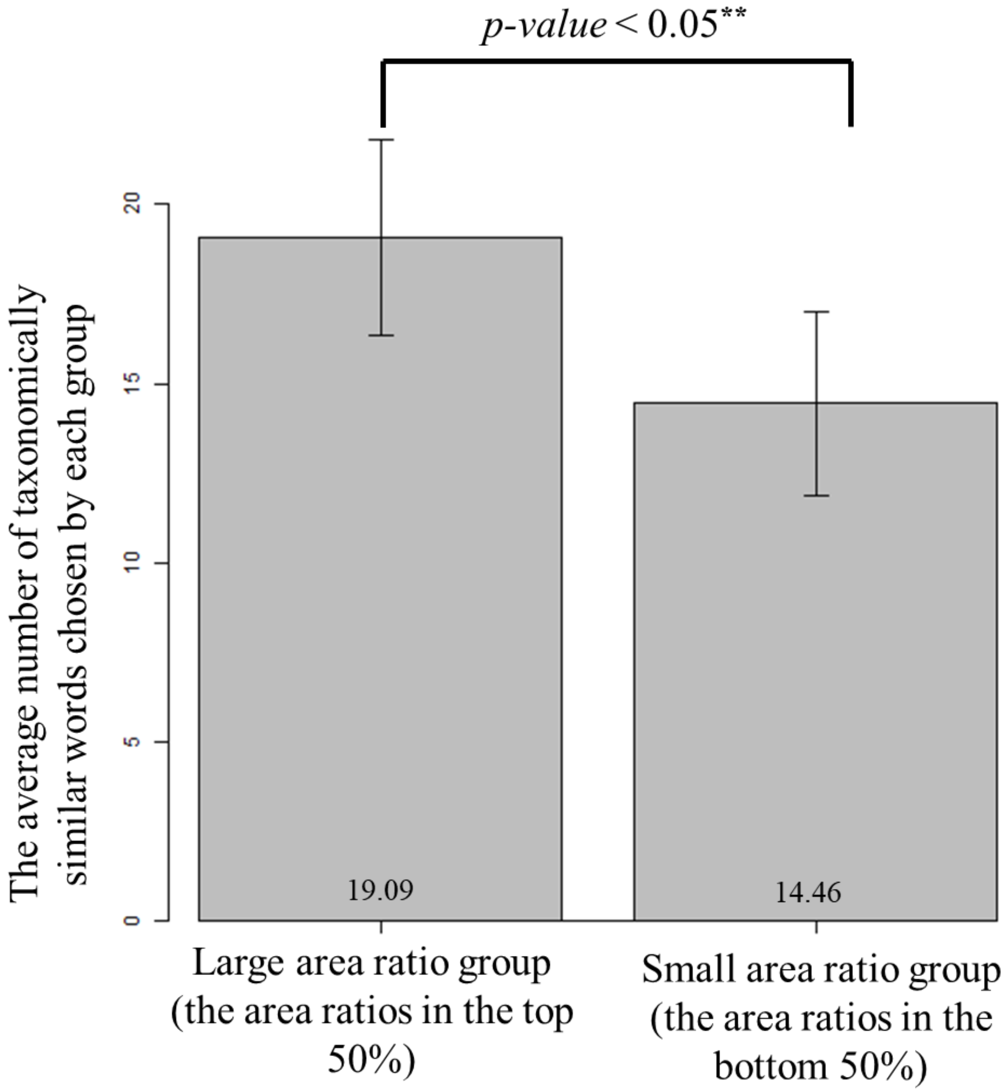

Fig. S2. Illustration of the relation between the average number of choosing taxonomically similar words and area ratio. The left bar shows the average number of taxonomically similar words chosen by the large area-ratio group (i.e., participants with area ratios in the top 50%) while the right bar, that of the small area-ratio group (i.e., participants with area ratios in the bottom 50%). The numbers inside the bars are average numbers.

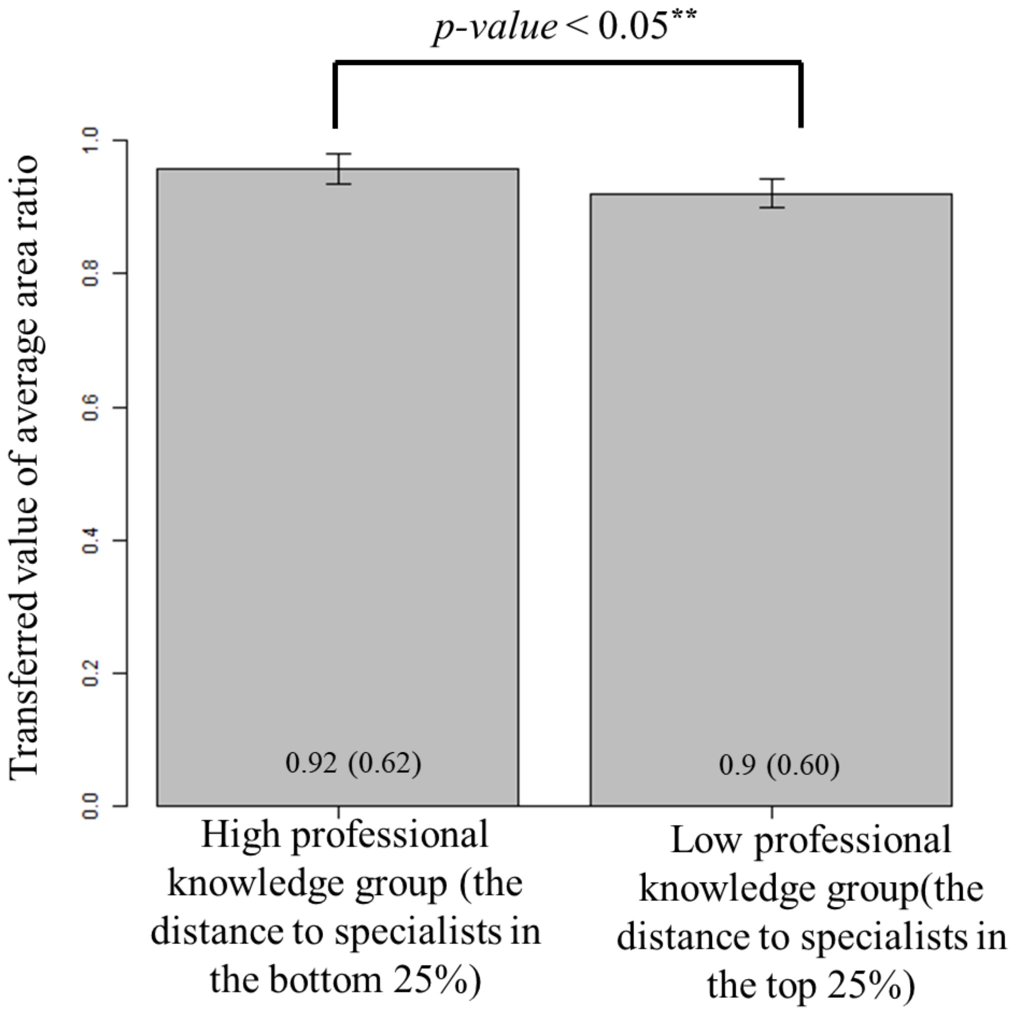

Fig. S3. Illustration of the average area ratio of the high and low professional knowledge groups in the Amazon review data. The left bar shows the average area-ratio of the high professional knowledge group (i.e., participants with distances to specialists in the bottom 25%) while the right bar, that of the low professional knowledge group (i.e., participants with distances to specialists in the top 25%). The numbers inside the bars are the transformed values of the average area ratios under the arcsine transformation. The numbers inside the parentheses are the raw average area ratios before the transformation.

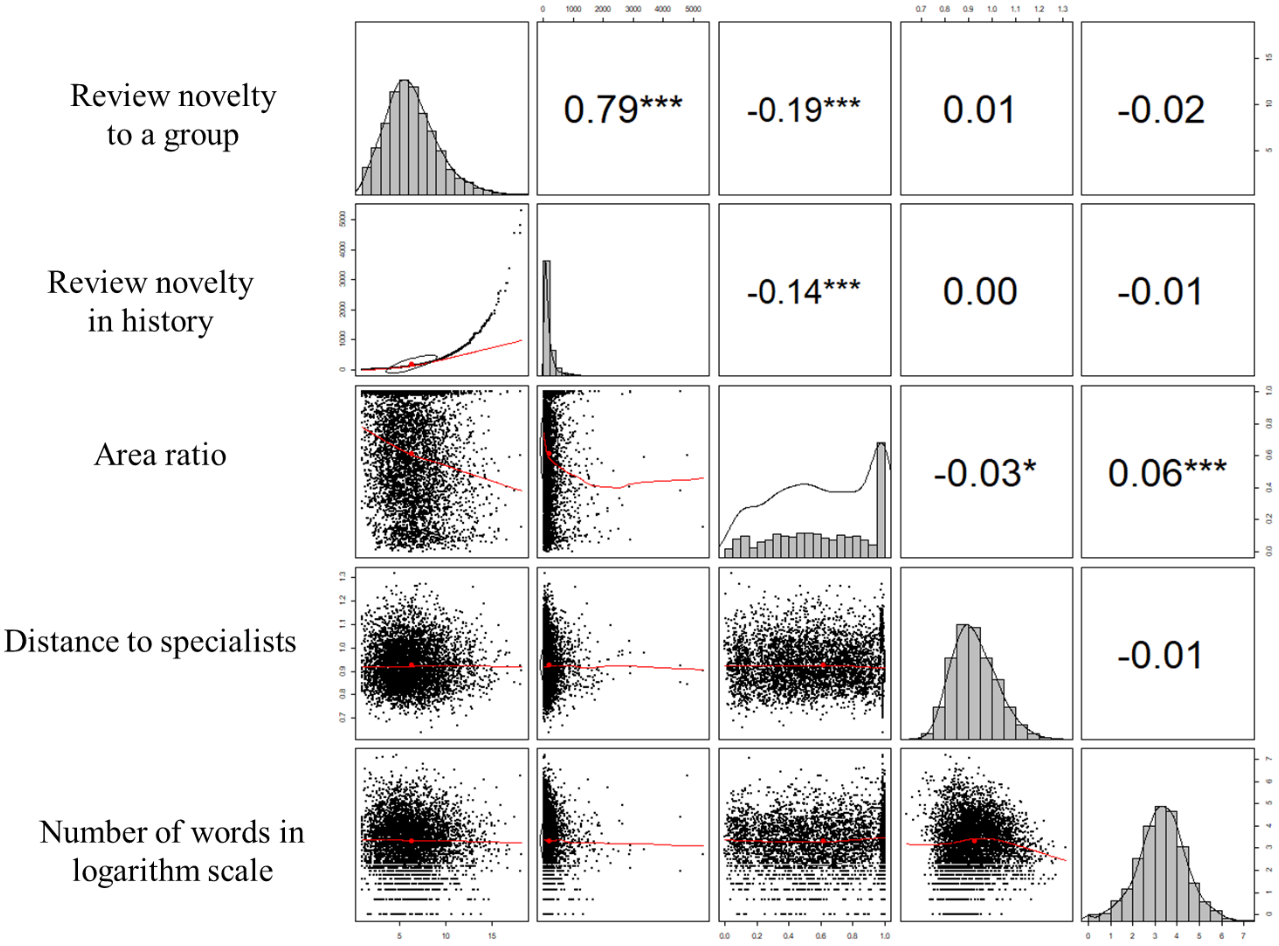

Fig. S4. Distribution, scatter plots, and correlations of all variables in regression models S1–S3. The names of the variables are on the left of the figure. In this figure, the diagonal shows the histograms, with the density curve of every variable. Graphs in the lower triangle show the scatter plots between each pair of variables. The red line shows the relation between these two variables predicted by linear regression (OLS). The ellipses show the correlation ellipses, with the centre of each as a red point. In the upper triangle, significant correlations between each pair of variables are shown. One asterisk refers to a *p*-value smaller than 0.1, two asterisks to a *p*-value smaller than 0.05, and three asterisks to a *p*-value smaller than 0.01.

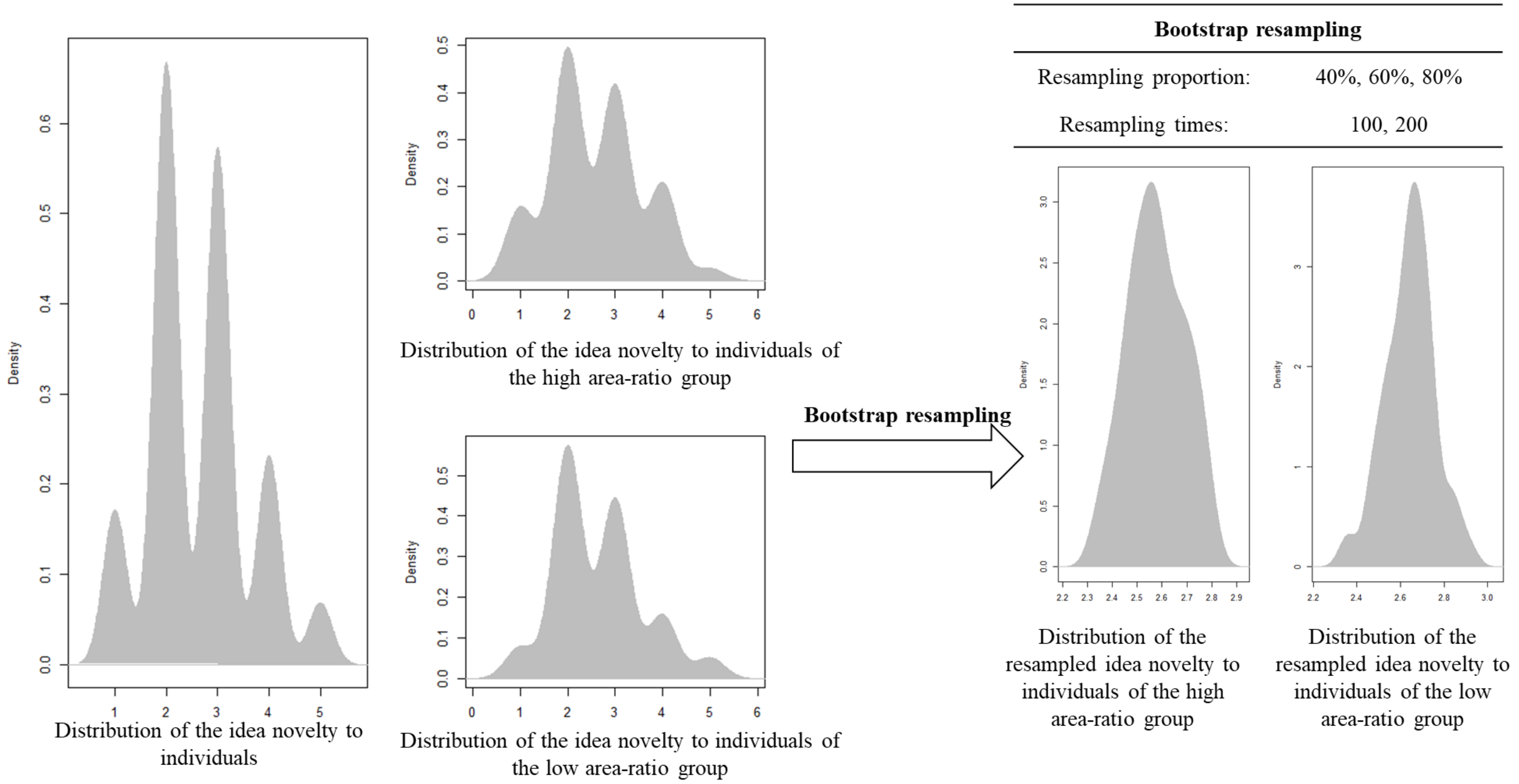

Fig. S5. Illustration of bootstrap resampling. The left panel shows the distributions of the idea novelty to individuals in the raw data and two groups under comparison (i.e., the high and low area-ratio groups in the study); the right panel shows the distributions of the resampled idea novelty to individuals in the two groups.

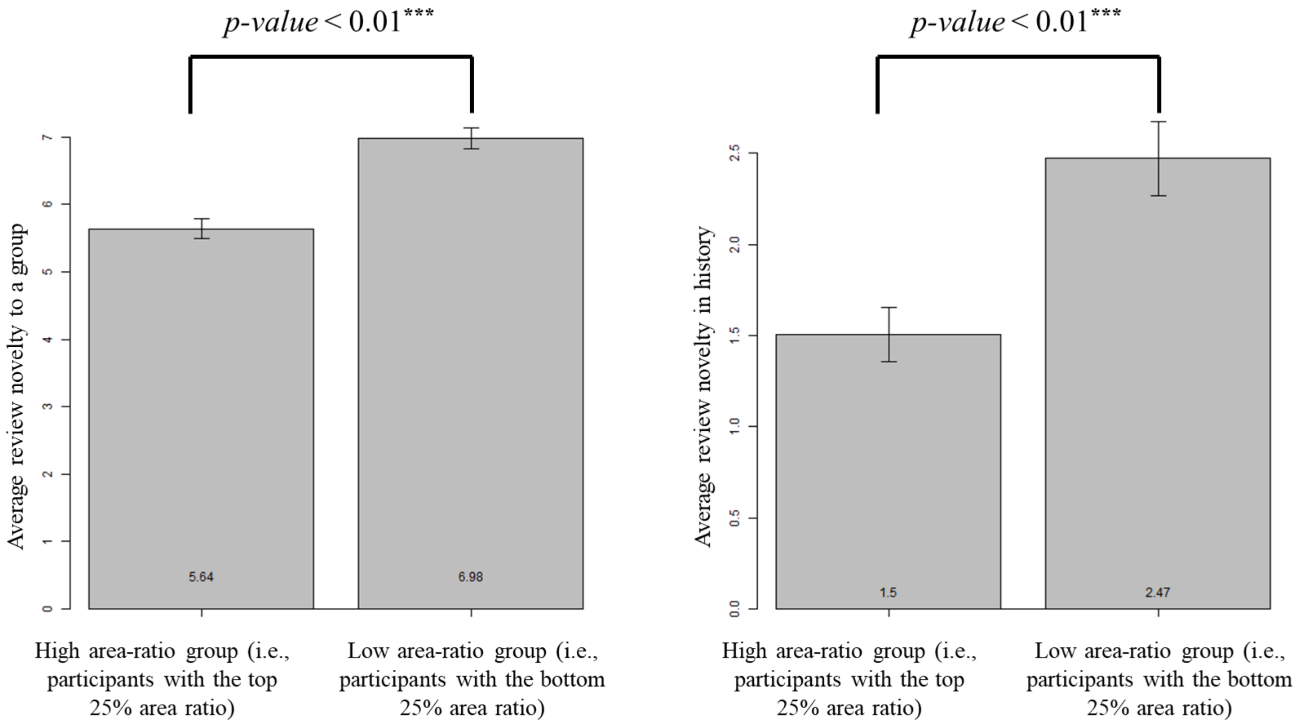

Fig. S6. Illustration of the impacts of attention deployment patterns on creative performances in the Amazon review data. The left panel shows the results based on the review novelty to a group while the right panel, based on the review novelty in history. The left bar of the bar chart in all panels indicates the average value of the metric in the high area-ratio group (i.e., participants with the top 25% area ratio) while the right bar, that of the low area-ratio group (i.e., participants with the bottom 25% area ratio).

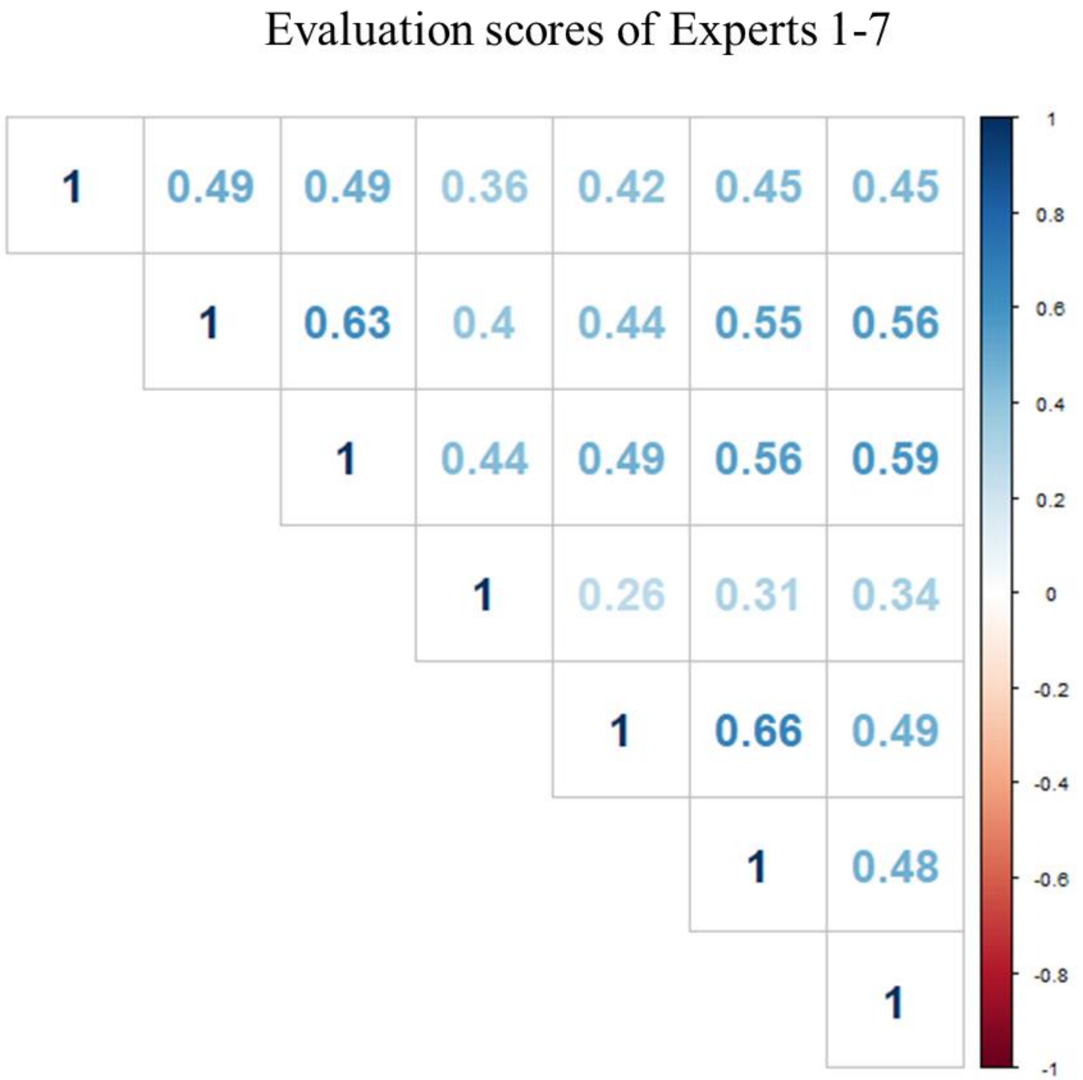

Fig. S7. Correlations among the evaluation scores of the seven experts. All correlations were significantly positive (with *p-value* < 0.05).

Table S1. Statistical information on all variables in regression models 1–5.

Statistic	Observations	Mean	*SD*	Min	Max
**Metrics of creative performances**					
Idea novelty to individuals	200	2.405	1.023	1	5
Idea novelty to a group	200	34.731	36.589	0.013	211.810
Idea novelty in history	200	36.849	28.964	0.207	151.872
**Metric of attention deployment patterns**					
Area ratio	200	0.301	0.178	0.001	0.859
**Metrics of regular users and specialists**					
Test score	200	8.955	4.587	0	20
**Control variables**					
Gender	200	1.140	0.348	1	2
Age	200	49.555	12.573	20	78
Number of speaker holdings	200	3.125	3.867	1	40
Frequency of speaker usage	200	5.245	1.786	1	7
Self-evaluation of the particularities of speakers	200	6.570	2.602	1	11
Self-evaluation of the amount of professional knowledge about speakers	200	4.785	2.536	1	11

Table S2. Statistical information on all variables in regression models S1–S3.

Statistic	Observations	Mean	*SD*	Min	Max
**Metrics of creative performances**					
Review novelty to a group	4,857	6.302	2.725	1.000	18.153
Review novelty in history	4,857	193.324	297.115	3.153	5,325.635
**Metrics of attention deployment patterns**					
Area ratio	4,857	0.613	0.307	0.001	1.000
**Metrics of regular users and specialists**					
Distance to specialists	4,857	0.926	0.094	0.637	1.315
**Control variable**					
Number of words in logarithm scale	4,857	3.306	1.096	0.000	7.175

Table S3. Regression results of the area ratio based on the Amazon review data.

Dependent variable	Area ratio
Model S1	
Distance to specialists	−0.961*** (0.189)
Number of words in the review (in logarithm scale)	0.083*** (0.016)
phi[1]	1.0*** (0.02)
Average constant[2]	1.354
Observations	4,857
R-square	0.007
Log Likelihood	3,644.402

Note: One asterisk refers to a *p*-value smaller than 0.1, two asterisks to a *p*-value smaller than 0.05, and three asterisks to a *p*-value smaller than 0.01; parentheses indicate the standard error of every variable.

[1] *phi* was estimated as a parameter that decided the shape of the beta-distribution for the models.

[2] Since the model estimated different constants for different product IDs, there were 187 different constants. The average value of these 187 constants is reported here.

Table S4. The robust test of the different idea novelties to individuals under different bootstrap resampling proportions and resampling times. The table shows the results of the statistical test on the comparison of idea novelty to an individual between the high and low area-ratio groups under different resampling proportions and resampling times. The row in blue shows the resampling proportion and resampling times to generate the results in Fig. 4 in the manuscript. The *p*-value shows the *p*-value of the difference between the two groups via *t*-test. The results are consistent under all different resampling proportions and resampling times.

Resampling proportion	Resampling times	Average idea novelty to individuals of high area-ratio group	Average idea novelty to individuals of low area-ratio group	t	p-value
60%	100	2.58	2.64	−4.12	$< 0.01^{***}$
60%	200	2.58	2.64	−5.19	$< 0.01^{***}$
40%	100	2.58	2.65	−3.09	$< 0.01^{***}$
40%	200	2.58	2.64	−3.20	$< 0.01^{***}$
80%	100	2.57	2.65	−8.88	$< 0.01^{***}$
80%	200	2.57	2.65	−10.76	$< 0.01^{***}$

Table S5. Regression results of the impact on creative performances based on the Amazon review data.

Dependent variable	Review novelty to a group	Review novelty in history
	Model S2	Model S3
Area ratio	−1.707*** (0.125)	−1.308*** (0.138)
Distance to specialists	0.047 (0.409)	-0.136 (0.450)
Number of words in logarithm scale	−0.033 (0.035)	−0.014 (0.039)
Constant	7.414*** (0.406)	2.906*** (0.447)
Observations	4,857	4,857
R-square/Log Likelihood	0.037	0.018

Note: One asterisk refers to a *p*-value smaller than 0.1, two asterisks to a *p*-value smaller than 0.05, and three asterisks to a *p*-value smaller than 0.01; parentheses indicate the standard error of every variable.

Table S6. Results of path analysis based on the survey data.

	Attention deployment pattern–Professional knowledge		
**Dependent variable**	**Area ratio**		
Test score	0.202^{***} (0.069)		
	**Creative performance–Attention deployment pattern + Professional knowledge**		
**Dependent variable**	**Idea novelty to individuals**	**Idea novelty to a group**	**Idea novelty in history**
Area ratio	-0.289^{***} (0.069)	-0.125^{*} (0.072)	-0.122^{*} (0.072)
Test score	0.027 (0.069)	0.047 (0.072)	0.031 (0.072)
Observations	200		

Note: One asterisk refers to a *p*-value smaller than 0.1, two asterisks to a *p*-value smaller than 0.05, and three asterisks to a *p*-value smaller than 0.01; parentheses indicate the standard error of every variable; the standard error of every coefficient is shown in '*()*.'

Table S7. Results of path analysis based on the Amazon review data.

	Attention deployment pattern–Professional knowledge	
**Dependent variable**	**Area Ratio**	
Distance to specialists	-0.029^{**} (0.014)	
	**Creative performance–Attention deployment pattern + Professional knowledge**	
**Dependent variable**	**Review novelty to a group**	**Review novelty in history**
Area ratio	-0.193^{***} (0.014)	-0.135^{***} (0.014)
Distance to specialists	0.002 (0.014)	-0.004 (0.014)
Observations	4,857	

Note: One asterisk refers to a *p*-value smaller than 0.1, two asterisks to a *p*-value smaller than 0.05, and three asterisks to a *p*-value smaller than 0.01; parentheses indicate the standard error of every variable; the standard error of every coefficient is shown in '*()*.'

SI References

1. Dean, D. L., Hender, J., Rodgers, T. & Santanen, E. Identifying good ideas: constructs and scales for idea evaluation. *J. Assoc. Inf. Syst.* **7**, 646–699 (2006).
2. Kudrowitz, B. M. & Wallace, D. Assessing the quality of ideas from prolific, early-stage product ideation. *J. Eng. Design* **24**, 120–139 (2013).
3. Tang, M., Gorelick, L., Veksler, O. & Boykov, Y. Grabcut in one cut. In *Proc. 2013 IEEE International Conference on Computer Vision* 1769–1776 (IEEE, 2013).
4. Huang, R., Gu, J., Sun, X., Hou, Y. & Uddin, S. A rapid recognition method for electronic components based on the improved YOLO-V3 network. *Electronics* **8**, 825–842 (2019).
5. Wang, J., Xiao, W. & Ni, T. Efficient object detection method based on improved YOLOv3 network for remote sensing images. In *Proc.3rd International Conference on Artificial Intelligence and Big Data* 242–246 (IEEE, 2020).
6. Ji, L-J., Zhang, Z. & Nisbett, R. E. Is it culture or is it language? Examination of language effects in cross-cultural research on categorization. *J. Pers. Soc. Psychol.* **87,** 57–65 (2004).
7. Simmons, S. & Estes, Z. Individual differences in the perception of similarity and difference. *Cognition* **108**, 781–795 (2008).
8. Nisbett, R. E. & Miyamoto, Y. The influence of culture: holistic versus analytic perception. *Trends Cogn. Sci.* **9**, 467–473 (2005).
9. Mirman, D., Landrigan, J-F. & Britt, A. E. Taxonomic and thematic semantic systems. *Psychol. Bull.* **143**, 499–543 (2017).

10. Burke, P. *A social history of knowledge II: From the encyclopaedia to Wikipedia* (Polity Press, 2012).

11. Bellomi, F. & Bonato, R. Network analysis for Wikipedia. In *Proc. Wikimania 2005,* (eds. Voss, J., Lih, A., Klein, S. & Ma, C.) 81–92 (Wikimeda Foundation, 2005).

12. Strube, M. & Ponzetto, S. P. WikiRelate! Computing semantic relatedness using Wikipedia. In *Proc. 21st National Conference on Artifical Intelligence* 1419–1424 (AAAI, 2006).

13. Silva, F. N., Viana, M. P., Travençolo, B. A. N. & Costa, L. F. Investigating relationships within and between category networks in Wikipedia. *J. Informetr.* **5**, 431–438 (2011).

14. Dean, D. L., Hender, J., Rodgers, T. & Santanen, E. Identifying good ideas: constructs and scales for idea evaluation. *J. Assoc. Inf. Syst.* **7**, 646–699 (2006).

15. Kudrowitz, B. M. & Wallace, D. Assessing the quality of ideas from prolific, early-stage product ideation. *J. Eng. Design* **24**, 120–139 (2013).

16. Kirstetter, E., Eagar, R., Kolk, M. & Roos, D. The creativity wra–a new paradigm for business. *Prism* **2013**, 12–29 (2013).

17. Ramos, J. Using tf-idf to determine word relevance in document queries. In *Proc. 12th International Conference on Machine Learning*, (eds. Fawcett, T. & Mishra, N.) 133–142 (AAAI, 2003).

18. Shi, F., Teplitskiy, M., Duede, E. & Evans, J. A. The wisdom of polarized crowds. *Nat. Hum. Behav.* **3**, 329–336 (2019).

19. Vilhena, D. A. *et al*. Finding cultural holes: how structure and culture diverge in

networks of scholarly communication. *Sociol. Sci.* **1**, 221–238 (2014).

20. Hogan, B. The presentation of self in the age of social media: distinguishing performances and exhibitions online. *Bull. Sci. Technol. Soc.* **30**, 377–386 (2010).
21. Cascio, M. T., Cella, M., Preti, A., Meneghelli, A. & Cocchi, A. gender and duration of untreated psychosis: a systematic review and meta-analysis. *Early Interv. Psychiatry* **6**, 115–127 (2012).
22. Fay, M. P. & Proschan, M. A. Wilcoxon-Mann-Whitney or t-test? On assumptions for hypothesis tests and multiple interpretations of decision rules. *Stat. Surv.* **4,** 1–39 (2010).
23. Lahiri, S. N. *Resampling methods for dependent data* (Springer Science & Business Media, 2013).
24. Zhao, X., Lynch, Jr. J. G. & Chen, Q. Reconsidering Baron and Kenny: myths and truths about mediation analysis. *J. Consum. Res.* **37**, 197–206 (2010).
